# Aspartame Endowed ZnO-Based Self-Healing Solid Electrolyte Interface Film for Long-Cycling and Wide-Temperature Aqueous Zn-Ion Batteries

**DOI:** 10.1007/s40820-025-01765-6

**Published:** 2025-05-12

**Authors:** Yunyu Shi, Yingkang Liu, Ruirui Chang, Guilin Zhang, Yuqing Rang, Zheng-Long Xu, Qi Meng, Penghui Cao, Xiangyang Zhou, Jingjing Tang, Juan Yang

**Affiliations:** 1 School of Metallurgy and Environment, Central South University, Changsha, 410083 People’s Republic of China; 2 Department of Industrial and Systems Engineering, Research Institute for Advanced Manufacturing, The Hong Kong Polytechnic University, Hung Hom, Hong Kong SAR People’s Republic of China; 3 College of Energy and Power Engineering, Changsha University of Science & Technology, Changsha, 410114 People’s Republic of China

**Keywords:** Aspartame additives, Self-healing ZnO-based SEI film, Long cycle life, Wide-temperature operation, Aqueous Zn-ion batteries

## Abstract

Metallic Zn anodes suffer from hydrogen evolution and dendritic deposition in aqueous electrolytes, resulting in low Coulombic efficiency and poor cyclic stability for aqueous Zn-ion batteries (AZIBs). Constructing stable solid electrolyte interphase (SEI) with strong affinity for Zn and exclusion of water corrosion of Zn metal anodes is a promising strategy to tackle these challenges. In this study, we develop a self-healing ZnO-based SEI film on the Zn electrode surface by employing an aspartame (APM) as a versatile electrolyte additive. The hydrophobic nature and strong Zn affinity of APM can facilitate the dynamic self-healing of ZnO-based SEI film during cyclic Zn plating/stripping process. Benefiting from the superior protection effect of self-healing ZnO-based SEI, the Zn║Cu cells possess an average coulombic efficiency more than 99.59% over 1,000 cycles even at a low current density of 1 mA cm^−2 ^− 1 mAh cm^−2^. Furthermore, the Zn║NH_4_^+^-V_2_O_5_ full cells display a large specific capacity of 150 mAh g^−1^ and high cyclic stability with a capacity retention of 77.8% after 1,750 cycles. In addition, the Zn║Zn cell delivers high temperature adaptability at a wide-temperature range from − 5 to 40 °C even under a high DOD of 85.2%. The enhanced capability and durability originate from the self-healing SEI formation enabled by multifunctional APM additives mediating both corrosion suppression and interfacial stabilization. This work presents an inspired and straightforward approach to promote a dendrite-free and wide-temperature rechargeable AZIBs energy storage system.

**Supplementary Information:**

The online version contains supplementary material available at 10.1007/s40820-025-01765-6.

## Highlights

- Aspartame additive in electrolyte enables the in situ formation of ZnO-based solid electrolyte interphase, enhancing Zn anode corrosion resistance and stability with excellent self-healing capabilities.
- Zn║Zn symmetric cells with APM-modified electrolyte operate stably for 6,400 h at − 5 °C, 10,330 h at 25 °C, and 2,250 h at 40 °C, with a high DOD of 85.2%.
- Achieves 99.59% Coulombic efficiency, suppresses dendrite growth, and maintains 150 mAh g^−1^ capacity after 1,750 cycles in NH^4+^-V_2_O_5_ full cells.

**Supplementary Information:**

The online version contains supplementary material available at 10.1007/s40820-025-01765-6.

## Graphical abstract

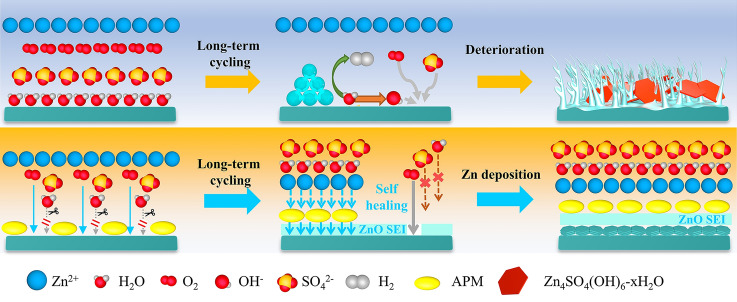

## Introduction

Aqueous Zn-ion batteries (AZIBs) are regarded as one of the most commercially viable options among the next-generation electrochemical energy storage systems. The high volumetric capacity and specific capacity of Zn metal endow AZIBs with unique advantages for large-scale applications [1–5]. However, the thermodynamic instability of Zn anode stemming from its low redox potential (− 0.762 V vs SHE) persistently compromises electrochemical performance through irreversible side reactions [6, 7]. The inevitable hydrogen evolution reaction (HER) severely depletes the electrolytes [8] and generates loose, poorly conductive by-products, such as Zn_4_(OH)_6_SO_4_·xH_2_O (ZSH) [9]. Dendritic Zn is further exacerbated by the enhanced “tip effect,” where Zn^2+^ deposition mostly happens at a limited number of highly active sites [10]. The aforementioned fundamental issues lead to an unstable electrolyte/electrode interface and the puncturing of separator, ultimately resulting in cell failure.

Recent advances in addressing the above critical issues for Zn anodes mainly contain the following three aspects [11, 12], including using hydrogels to restrict the migration of free H_2_O [13, 14], modifying the Zn metal surface [15–17], and limiting harmful ion transport through the separator [18, 19]. Among them, electrolyte engineering is considered as one of the most straightforward and effective strategies to stabilize Zn electrode, which can directly change the deposition behavior of Zn^2+^ and affect the interface properties of Zn electrode [20, 21]. The common electrolyte modification methods include altering the solvation structure of Zn^2+^ [22–25], modifying the hydrogen bonding network of H_2_O molecules [26, 27], adjusting electric field distribution at the interface [28–30], and regulating the inner Helmholtz plane (IHP) [31, 32]. Previous research on electrolyte additives primarily concentrated on the bulk phase of the electrolyte and the interface between electrolyte and Zn electrode to mitigate Zn corrosion caused by the aqueous electrolyte [33, 34]. The effects of other solutes in the electrolyte except H_2_O and electrolyte salts on Zn metal are always ignored. However, because the anion decomposition voltage in the electrolyte is much higher than that in H_2_O, SEI formation on the surface of the Zn electrode is less studied [35]. Therefore, constructing SEI film with the ability of corrosion resistance and self-healing on the surface of Zn electrode via facile method will be of great significance for realizing the long-life AZIBs.

Several strategies have been proposed to construct SEI artificially, such as missing linker bifunctional C_48_H_24_O_36_Ti_8_ (MIL-125-Ti) [36], etching coating in methyl triethoxysilane and H_2_ZrF_6_ [37], amorphous MOF [38], etc. Artificial SEI films produced by scraping or spin coating can form a uniform protective barrier, and up to now, a wide variety of coatings and coating methods were proposed to provide a strong hydrophobic barrier and a means of Zn^2+^ transport [39–41]. However, the artificial constructed coatings tend to have a weak bond with the Zn metal, resulting in falling off within short-term cycles. On the contrary, the *in situ*-formed SEI chemically anchored to the Zn electrode exhibits robust interfacial adhesion, effectively mitigating delamination phenomena during electrochemical cycling [42].

The current methods for SEI construction are mainly achieved by introducing some additives that can self-polymerize [43] or react with Zn electrode [44], among which the most potential is to induce the construction of ZnO SEI, which possesses excellent electronic insulating properties and high ionic conduction rate on the surface of Zn electrode [45, 46]. Nonetheless, some critical issues of ZnO-based SEI are still unsettled, such as the weak adhesion of ZnO-based SEI on Zn electrode during repeated Zn plating/stripping, the decomposition of SEI in electrolyte, inability of ZnO to maintain stability through self-healing, and so on [35]. These factors lead to rapid failure of the SEI film during cycling and increased polarization voltage. Therefore, it is essential to develop a straightforward method for constructing *in situ* SEI that enhances the corrosion resistance of Zn electrode, possesses self-healing capabilities, and maintains integrity during cycling. Additionally, the SEI should exhibit fast ionic conductivity to ensure rapid Zn^2+^ conduction kinetics.

In this work, we successfully constructed a self-healing ZnO-based SEI film on the surface of Zn anode by introducing an appropriate amount of aspartame (APM) into the electrolyte of AZIBs. Density function theory (DFT) calculations and molecular dynamics simulations (MDS) demonstrate that APM can preferentially adsorb at the Zn/electrolyte interface and isolate H_2_O molecules and SO_4_^2−^ anions from the Zn electrode. The adsorbed APM can react with the dissolved O_2_ to generate a ZnO-based SEI film on the Zn electrode with self-healing ability and strong chemical connection with the Zn substrate. Owing to the dynamically stable SEI film, the Zn║Zn symmetric cells demonstrate exceptional temperature adaptability (− 10 to 40 °C) and enhanced calendar longevity under stringent deep discharge conditions (85% DOD). This work provides an environmentally friendly and cost-effective electrolyte additive, offering a viable strategy for the *in situ* generation of SEI film on Zn anode. The stable and robust ZnO-based SEI film significantly enhances the performance of AZIBs, offering valuable new insights into the interface challenges of SEI formation in aqueous electrolytes on Zn electrode.

## Experimental Section

### Materials

Zinc sulfate heptahydrate (ZnSO_4_, AR) and zinc oxide (ZnO, AR) were obtained from Sinopharm, aspartame powder with purity > 98% was purchased from Aladdin, and ammonium metavanadate (NH_4_VO_3_, AR), zinc trifluoromethane sulfonate (Zn(OTf)_2_, purity > 98%), and zinc bromide (ZnBr_2_, purity > 98%) were purchased from Aladdin. High-purity Zn foil (50 μm, 99.99%) was purchased from Kintek Solution Ltd.

### Material Preparation and Cell Assembly

#### Preparations of the Anodes

The Zn foil was immersed in a 0.1 M HCl aqueous solution for 0.5 h to remove surface oil residues and impurities.

#### Preparations of the Electrolytes

2 M ZnSO_4_ in DI water was prepared as original electrolyte and denoted as BE. And then, various amounts of aspartame were added in BE for uniformed dissolution to achieve APM-BE electrolyte with different APM concentrations (0.5, 1.0, 2.0, 4.0, and 8.0 mg mL^−1^). To confirm the wide-ranging effectiveness of the APM additive, similar experiments were conducted using 2 M solutions of Zn(OTf)_2_ and ZnBr_2_. All chemicals were used as received without further purification.

#### Preparations of the Cathode Materials

The NH_4_-V_2_O_5_ cathode material was synthesized through a process of thermal decomposition. Typically, 1 g of NH_4_VO_3_ powder was carefully measured and heated at 300 °C for 2 h with a heating rate of 5 °C min^−1^. The obtained NH_4_-V_2_O_5_ powder was ready for subsequent applications.

#### Cell Assembly

CR2025 cells were used in this study. The Zn║Zn symmetric cells were assembled using Zn foils (thickness: 50 μm; diameter: 14 mm) as the electrodes and commercial Whatman glass fiber as the separator (thickness: 500 μm; diameter: 19 mm). Cu foils (thickness: 20 μm; diameter: 14 mm) and Ti foils (thickness: 20 μm; diameter: 14 mm) were also used in Zn║Cu and Zn║Ti asymmetric cells, respectively. For the Zn║NH_4_^+^-V_2_O_5_ full cell, the cathodes were prepared by mixing 70 wt% active material (NH_4_^+^-V_2_O_5_), 20 wt% conductive carbon (Super P), and 10 wt% polyvinylidene difluoride (PVDF) in N-methyl-2-pyrrolidone (NMP). The mixed homogeneous slurry was cast on stainless steel mesh by doctor blading. After drying at 80 °C overnight, the electrodes were punched into 10 mm disks for use. The zinc oxide cathode electrode sheet is prepared using the same method, except that vanadium pentoxide is replaced with zinc oxide.

### Materials Characterization

X-ray diffraction (XRD) was carried out with a Panalytical Empyrean-type X-ray diffractometer (Cu Kα) to analyze the crystal structure and composition of the Zn anode. Scanning electron microscopy (SEM, MIRA4 LMH) was used to observe the morphology of the surface and cross section of the Zn anode under different experimental conditions, and the elemental distribution on the surface was analyzed using an accompanying energy-dispersive spectrometer (EDS, Ultim Max 40). Focused ion beam (FIB) depth analysis was conducted using a Helios G4 PFIB HXe system. The X-ray photoelectron spectroscopy (XPS) was measured with an X-ray photoelectron spectrometer (Thermo Scientific K-Alpha) equipped with an Al Kα X-ray source (1486.6 eV) and an argon ion etching device to reflect elemental information on the surface and in the depth direction. Atomic force microscopy (AFM) was used to construct 3D images on zinc negative electrodes after charge/discharge cycling to characterize the surface roughness of electrodes. Fourier transform infrared spectroscopy (FTIR, Thermo Scientific, Nicolet iS50) was recorded in the 400–4000 cm^−1^ wavelength range. Raman spectra were measured by a Raman spectrometer (Horiba LabRAM HR Evolution) with a laser wavelength of 785 nm in the wave number range 50–4000 cm^−1^. The wettability of the electrolyte on the surface of the Zn anode was tested by means of a contact angle measuring instrument (Lauda Scientific LSA100). The nuclear magnetic resonance (NMR) spectrometer used in this study was a Bruker AV II-600 MHz from Bruker Corporation, Switzerland. Differential scanning calorimetry (DSC) was performed using a WITec alpha300R. *In situ* electrochemical microscopy (NSZ-808, CT2001A) was used to observe the growth of zinc dendrites at the interface between the zinc anode and the electrolyte at a current density of 5 mA cm^−2^. The micromorphology, crystal structure, and composition of the deposits on the surface of the Zn anode were investigated using a transmission electron microscope (TEM, JEM-F200) at a voltage of 200 kV and an accompanying energy-dispersive X-ray spectrometer. Regarding the TEM analysis of the SEI film on the Zn anode surface, we performed slight surface processing on the cycled Zn electrode using a file and analyzed the removed material via TEM. The pH of different solutions was measured using the Lichen PH-100 tester.

### Electrochemical Measurements

The electrochemical performances of cells were tested on the LAND testing system. The tests of electrochemical impedance spectroscopy (EIS), linear sweep voltammetry (LSV), chronoamperometry (CA), Tafel plots, and cyclic voltammetry (CV) were conducted on PARSTAT 4000 electrochemical workstation. Specifically, EIS for Zn║Zn symmetric cells and Zn║NH_4_^+^-V_2_O_5_ full cells was available in the frequency range of 100 kHz to 0.01 Hz. The overvoltage of chronoamperometry measurement was set to -150 mV, and a three-electrode system was used for the study. LSV was performed at a scan rate of 1 mV s^−1^ using Zn║Ti asymmetric cells. Tafel was measured at a scanning rate of 0.5 mV s^−1^ with a potential range of ± 0.25 V versus open-circuit potential. The working, reference, and auxiliary electrodes were zinc foil, Ag/AgCl, and platinum plate. The scanning rates of the CV curves were 0.1 and 1 mV s^−1^ for the full cell and symmetric cell, respectively.

The ionic conductivity of the electrolyte was obtained by performing EIS tests on cells with blocking electrode (stainless steel, SS) and is calculated by Eq. 1:

1 $$\sigma = \frac{l}{{{\text{SR}}_{S} }}$$

where σ (mS cm^−1^) represents the ionic conductivity of the electrolyte, *l* (cm) represents the distance between the electrodes, *R*_*S*_ (ohm) represents the solution resistance obtained through the EIS of the symmetric stainless steel cell. *S* (cm^−2^) represents the electrode area.

The activation energies of different types of electrolytes were obtained by performing EIS tests on Zn║Zn symmetric cells at different temperatures and are calculated by Eq. 2:

2 $$\frac{1}{{R_{{{\text{ct}}}} }} = A\exp (\frac{{{-}E_{a} }}{{{\text{RT}}}})$$

where *E*_a_, *R*_ct_, A, R, and *T* represent the desolvation energy, charge transfer resistance, the frequency factor, the gas constant, and the absolute temperature, respectively.

The depth of discharge of a Zn║Zn symmetric cell is calculated by combining two parameters, the surface capacity applied to the cell, and the thickness of the Zn electrodes, with Eq. 3:

3 $$\text{DOD} = \frac{{\text{c}}_{\text{a}}}{{{\text{h}}_{0}{\text{c}}}_{\text{v}}}$$

where *c*_*a*_ (mAh cm^−2^) is the surface capacity set in the measured conditions, *c*_*v*_ (mAh cm^−3^) is the volumetric specific capacity of the Zn metal (5855 mAh cm^−3^)*, h*_*o*_(cm)is the thickness of the Zn electrode.

The Gibbs free energy (ΔG) of the dehydration reaction between Zn(OH)_2_ and APM is calculated using Eq. 4:

4 $$\text{Einteraction} = \text{EAB}+\text{EBSSE}-EA-\text{EB}$$

where Einteraction is the interaction energy of the hydrogen bond and E_AB_ is the energy of the complex formed by the hydrogen bond interaction. E_A_ and EB are the energies of the molecule providing HD and HA, respectively. E_BSSE_ is the basis set superposition error (BSSE) that should be corrected when calculating the weak interaction.

The N/P ratio of the full cell is calculated using Eq. 5:

5 $$\frac{\text{N}}{{\text{P}}}\text{=}\frac{{\text{Q}}_{\text{Zinc Anode Capacity}}}{{\text{Q}}_{\text{Vanadium Pentoxide Cathode Capacity}}}\text{=}\frac{{\text{Q}}_{\text{theoretical capacity of zinc}}\text{*}{\text{m}}_{\text{zinc}}}{{\text{Q}}_{\text{theoretical capacity of vanadium pentoxide}}\text{*}{\text{m}}_{\text{vanadium pentoxide}}}$$

Among these, the theoretical specific capacity of zinc is 810 mAh g^−1^, while that of vanadium pentoxide (V_2_O_5_) is 589 mAh g^−1^. The mass of zinc is 0.011 g, and the N/P ratio is controlled by adjusting the cathode loading.

### Simulation and Theoretical Calculation

#### Molecular Dynamics (MD) Simulations

Simulations were carried out using the Materials Studio software, where the universal force field was used to simulate the molecular dynamics process [47]. The time step in molecular dynamics was 0.5 fs. In the equilibrium stage, the system was equilibrated at 298 K for 100 ps in the NPT ensemble. Then molecular dynamics was performed at 298 K for 500 ps in the NVT ensemble.

#### Density Functional Theory (DFT) Calculation

The study of hydrogen bonds formed between APM and different substances was calculated by Gaussian 16 software package [48]. The Geometrical optimization and frequency analysis were performed at M06-2X density functional and the def2-SVP basis set [8, 49, 50]. The electrostatic potential (ESP) was obtained by Multiwfn and VMD software package [51]. The interaction energy of a hydrogen bond is defined by Eq. 4.

The process of modeling started from hexagonal Zn cell (space group: P63/mmc) as shown in Fig. S7a. Two Zn surfaces were built whose exposed crystal face corresponds to the strongest XRD peaks (100) and (002) as shown in Fig. S7b, c. Given the bulkiness of aspartame and the number of atom in whole structure, two Zn slabs with a thickness of about 8 Å [three films] were constructed in two supercell [8 × 8 for Zn (002) and 8 × 4 for Zn (100)] containing a vacuum film (more than 15 Å). The entire structures of Zn surface consist 192 atoms, respectively.

K-point sampling was carried out using (1, 1, 1) Gamma grids, respectively. Structural relaxations were conducted employing conjugate gradient optimization, with the Hellmann–Feynman theorem utilized for force calculations.

The first-principles work was performed using the DFT as implemented in Vienna Ab-initio Simulation Package (VASP). The Kohn–Sham orbitals were expanded using plane waves with a kinetic energy cutoff of 550 eV. A conjugate gradient algorithm was used to relax the ions into their instantaneous ground state. The projector-augmented wave method was used to describe the interaction between valence electrons and the core. The projection scheme, integral to the functionality of the PAW method, was determined through fully automatic optimization of projection operators. The PBE functional was used to treat the exchange–correlation at the level of non-spin-polarized generalized gradient approximation. The partial occupancies f_nk_ were set for each orbital by using Gaussian smearing. The convergence conditions for the total energy change and force were less than 1 × 10^–6^ eV and 0.05 eV Å^−1^, respectively.

## Results and Discussion

### Effect of APM Molecule on the Electrolyte Phase and the Interface Between Zn Electrode and Electrolyte

**Fig. 1:**
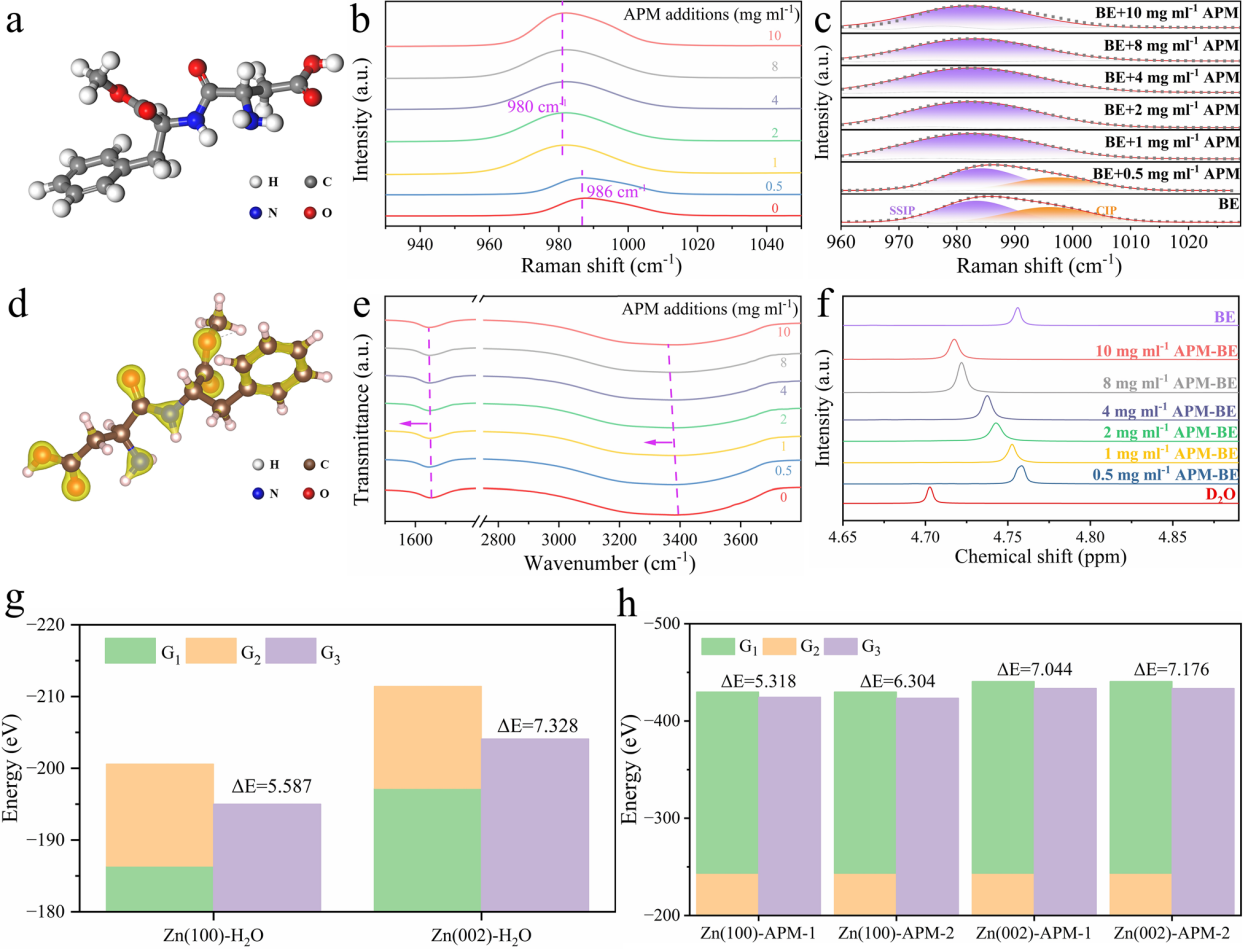
Physicochemical and Interfacial Characteristics of APM-Modified Electrolytes. **a** Structural map of APM molecule. **b** Raman spectra of electrolyte with varying APM concentrations and **c** the peak fitting results of sulfate radical vibration peaks. **d** Charge density distribution of APM (Isosurface level:0.27). **e** FTIR and **f** H NMR spectra of electrolyte with varying APM concentrations. Calculated adsorption energies of **g** APM and **h** H_2_O molecules with Zn electrode based on Table S1

The properties of the electrolytic liquid phase and interface are very important for the stability of Zn anodes. The APM molecule (Fig. 1a) contains many polar groups and spectrum was carried out to explore influence of APM on the phase and interface. Firstly, electrolytes with different APM addition concentrations were analyzed using Raman spectroscopy and FTIR. As shown in Fig. 1b, c, the traditional Eigen–Tamm (ET) mechanism classifies ion-pair species into two main categories: solvation-separated ion pairs (SSIP), represented as [Zn^2+^-(H_2_O)_n_·SO_4_^2−^], and contact ion pairs (CIP), denoted as [Zn^2+^-(H_2_O)_n−1_·OSO_3_^2−^] (Fig. S1). Different concentrations of APM additives can regulate the relative contributions of the two ion pairs mentioned above. In BE, the proportion of SSIP and CIP, determined from the peak area ratio, is 55.3% and 44.7%, respectively. As the APM concentration increases, the fraction of SSIP gradually rises until CIP is completely eliminated, indicating that SO_4_^2−^ struggles to penetrate the inner film of the solvation shell [52–54]. The radial distribution function (RDF) of SO_4_^2−^ and H_2_O molecules in different types of electrolytes also shows a significant decrease in the sharp peak at 2.83 Å and coordination number (CN) in APM-BE (Fig. S2), which is consistent with the results of Raman spectroscopy.

To elucidate the hydrogen bonding interactions between APM and H_2_O, Fourier transform infrared (FTIR) was performed on the electrolytes with APM concentrations ranging from 0 to 10 mg mL^−1^. As shown in Fig. 1e, the prominent peaks in the regions of 2800 ~ 3800 and 1500 ~ 1750 cm^−1^ are ascribed to the O–H stretching vibration and the bending vibrational modes of the H_2_O molecule, respectively. These vibrations models are highly sensitive to the formation of hydrogen bonds [27]. With the increasing concentration of APM, the O–H bending and stretching vibration peaks of H_2_O molecules shift concurrently to lower wavenumbers, indicating that the O–H force is constant and the bond energy gradually decreases. Next, NMR testing was used to analyze the interaction between zinc ions and water molecules in electrolytes with different APM concentrations. The results showed that the NMR characteristic peaks of D_2_O and BE were located at 4.702 and 4.761 ppm, respectively. The shift of the water molecule peak to a higher frequency indicates a decrease in electron density, suggesting a strong interaction between Zn^2+^ and water molecules. As the APM concentration gradually increased, the characteristic peak of water molecules shifted progressively to a lower frequency, indicating an increase in the electron cloud density around water molecules and a weakened interaction between Zn^2+^ and water [55] (Fig. 1f). The experimental results demonstrate that the APM additive is capable of forming hydrogen bonds with H_2_O molecules. It not only diminishes the SO_4_^2−^ content within the solvation structure of Zn^2+^, but also restricts the free movement of H_2_O molecules through hydrogen bonding [56, 57].

The interfacial properties between the electrolyte and the anode also affect the stability of the Zn electrode, and the contact angle can characterize the interactions between electrolyte molecules and the electrode on a macroscopic scale. Therefore, it is necessary to study the contact angles of electrolytes with different APM concentrations on the Zn electrode. The results of the wettability of the above-mentioned electrolyte with the Zn metal indicate that with the increase in APM concentration, the contact angle decreases first and then increases [58] (Fig. S3). The XPS of the Zn electrode after cycling in APM-BE showed strong peaks for C-O (289.4 eV) and N–H (400.0 eV), whereas the energy spectra of the Zn electrode in BE after cycling did not show such peaks (Fig. S4), which strongly supports the characteristic adsorption of APM on the Zn electrode. This demonstrates that this variation in contact angle is attributed to the competition between the water content in the EDL and the adsorption capacity of APM. At low APM concentrations, the strong adsorption of APM at the Zn electrode interface plays a more dominant role in reducing the contact angle compared to the decrease in water content, leading to an overall decrease in the contact angle. However, with the continuous increase in APM adsorption, the substantial depletion of water content in the EDL eventually becomes the dominant factor. At this stage, the adsorption capacity of APM is no longer sufficient to counterbalance this effect, ultimately leading to a further increase in contact angle. In order to evaluate the concentration-dependent effect of APM on electrolyte conductivity, symmetric SS║SS cells were assembled for EIS measurements. The electrolyte conductivities were determined via equivalent circuit fitting of EIS curves and are calculated using Eq. 1. Notably, at higher concentration, APM induces a pronounced reduction in electrolyte conductivities (Fig. S5), which consequently results in a significant degradation of electrochemical performance. Hence, 1 mg mL^−1^ was selected as the optimal concentration of APM additive for the subsequent discussion.

The interfacial properties between Zn electrode and electrolyte are considered to be a key factor influencing the electrochemical performance of AZIBs [59]. DFT was used to calculate the adsorption energy of APM on various Zn crystal faces. Results show that on the APM molecules, charge density is more concentrated near the highly electronegative N (blue) and O (red) atoms (Isosurface level: 0.27) (Fig. 1d). Additionally, the benzene ring in APM also shows higher charge density. The amino, carboxyl, and carbonyl groups, along with the benzene ring, primarily exhibit electron-withdrawing properties. This characteristic promotes interaction between APM and the electron-rich metal surface, strengthening interfacial adsorption and enhancing the corrosion resistance of the Zn anode.

In order to investigate the adsorption behavior of water molecules and APM additives on the surface of Zn electrode, DFT simulated their adsorption process on the surface of Zn electrode. To model the adsorption of molecules on the Zn electrode surface, the adsorbed H_2_O or APM is initially positioned above the metal surface (Fig. S6). Given the bulkiness of APM molecules, two orientations of APM molecules are incorporated into the construction of the adsorption model. After the relaxation, the adsorption energies of all cases are presented in Table S1. As shown in Fig. 1g, h, the Zn (002) plane exhibits a lower total crystal plane energy (10.829 eV) compared to the Zn (100) plane (0.056 eV atom^−1^). The energy of adsorption states of the two adsorbed molecules on Zn (002) is lower than that on Zn (100), indicating Zn (002) is more suitable for APM adsorption. In contrast, APM molecules have higher adsorption energy than H_2_O on both Zn (100) (5.587 vs. 5.318 eV) or Zn (002) (7.328 vs. 7.044 eV) surfaces (except for Zn100-APM-2), indicating the preferential adsorption of APM molecules on these Zn crystal planes.

Additionally, the adsorption energy varies with different orientations of APM molecules during adsorption. In order to investigate the dominant configuration of APM molecules during adsorption, two typical configurations were selected for analysis. Specifically, the adsorption energy of Zn (002) and (001) -APM-1 (5.318 and 7.044 eV) is lower than that of Zn (002) and (001) -APM-2 (6.304 and 7.176 eV). The impact of molecular orientation on adsorption energy is revealed by the 2D charge density (Fig. S8). The optimization results from Zn(002)-APM-2 indicate significant molecular rotation due to relaxation processes (Fig. S9), resulting in an increased distance between the benzene ring and the metal surface. This suggests that the benzene ring exhibits weaker attraction to the electrons on the metal surface compared to the N and O atoms. Therefore, O and N atoms in the APM molecule serve as partial electron-donating centers. Their proximity to the Zn electrode allows for stronger interactions with the Zn atoms, making adsorption easier and the structure more stable.

### Morphology and Composition Analysis of Zn Electrode Surface and ZnO-Based SEI

**Fig. 2:**
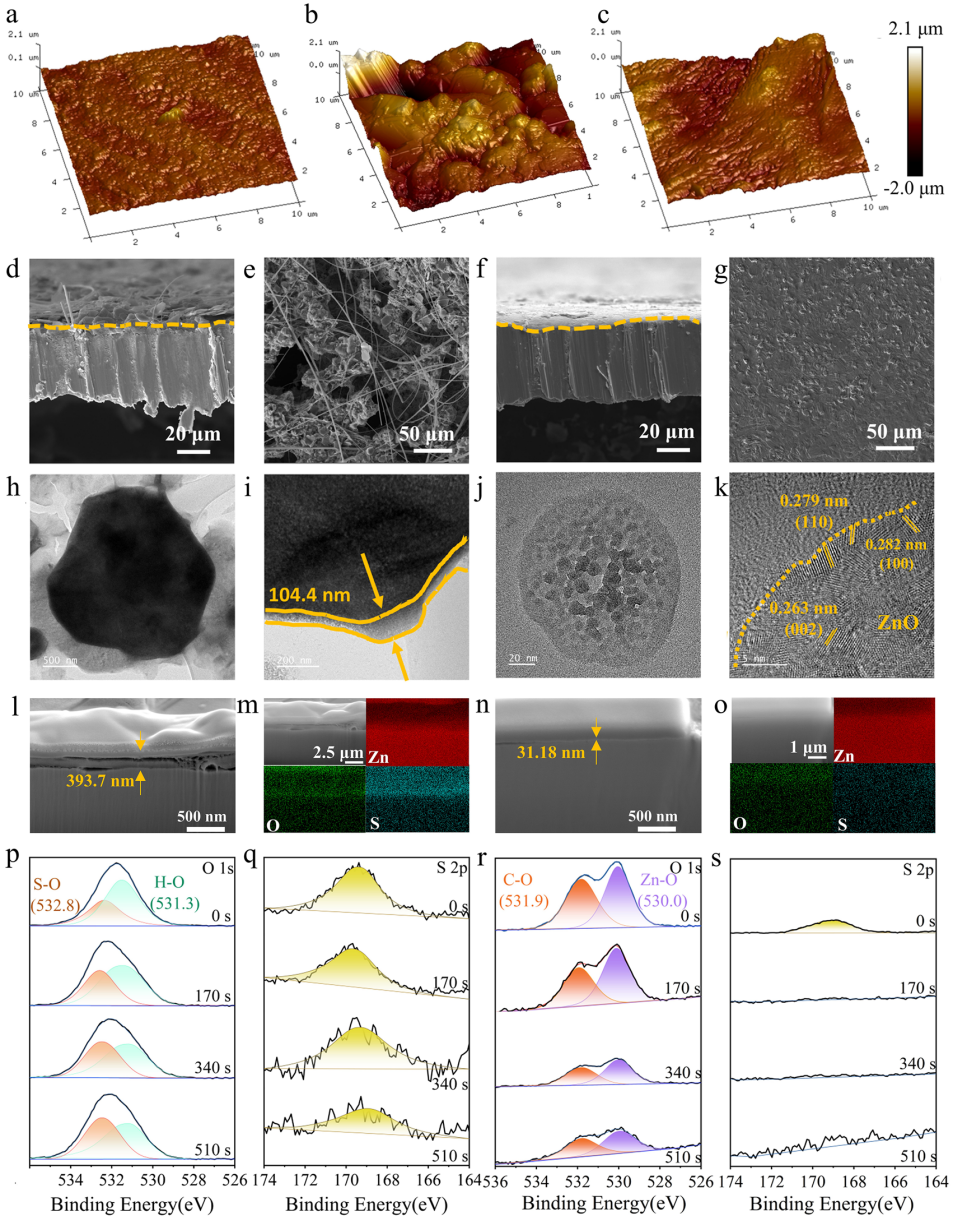
Morphology and composition characterizations of Zn electrode. AFM scanning patterns of the Zn electrode: **a** uncycled, after 50 cycles **b** in BE and **c** in APM-BE. SEM images of cross sections and surfaces of Zn electrode cycling in **d–e** BE and **f–g** APM-BE. **h** TEM images of the SEI of Zn electrode cycling in BE. **i** HRTEM images of the deposits. **j–k** TEM images of the surface deposits of Zn electrode cycling in APM-BE. **l** FIB cross-sectional SEM images and **m** EDS mapping images of Zn electrode after cycling in BE. **n** FIB cross-sectional SEM images and **o** EDS mapping images of Zn electrode after cycling in APM-BE. XPS depth tests of Zn electrode in **p–q** BE and **r–s** APM-BE. All electrodes employed in the aforementioned characterizations were obtained after 50 cycles at 5 mA cm^−2^–5 mAh cm^−2^

To investigate the influence of APM on the surface architecture of Zn anode in AZIBs and its regulatory effect on SEI formation during electrochemical cycling, we performed constant current cycling tests using Zn║Zn symmetric cells for systematic characterization of the cycled electrodes' structural evolution, compositional changes, and surface chemical bonding states. AFM analysis of the cycled Zn electrodes revealed significant differences. Specifically, the R_q_ of the uncycled Zn foil is 101 nm; the surface of Zn electrolyte cycled in APM-BE demonstrated a lower surface roughness (R_q_ = 151 nm) compared to that observed in conventional BE (R_q_ = 560 nm) (Fig. 2a-c). Furthermore, the Zn electrode cycled in APM-BE exhibited a smoother surface morphology compared to that cycled in conventional BE. As illustrated in Fig. 2d–g, the Zn electrode cycled in BE displayed pronounced stripped separator and pores, whereas the APM-BE yielded a more uniform and planar surface structure. Additionally, XRD analysis showed significant variations in Zn_4_(OH)_6_SO_4_·xH_2_O by-product growth between the two samples (Fig. S10). These observations indicate that the Zn plating/stripping processes are more homogeneously in the APM-BE system. The phase composition of the cycled Zn electrode surface was analyzed using TEM, revealing the interfacial evolution of the Zn electrode during cycling. After cycling in BE, the Zn hexagonal platelets are covered with a film of Zn_4_(OH)_6_SO_4_·xH_2_O by-products with a thickness of more than 100 nm (Fig. 2h, i), which severely imped the deposition kinetics of Zn^2+^. Moreover, the passivation film formed by Zn_4_(OH)_6_SO_4_·xH_2_O has a crystal structure only in a very small area (Fig. S13b), as evidenced by the coarse diffraction rings in its selected-area electron diffraction (SAED). Additionally, FIB-SEM depth analysis of the Zn electrode cycled in BE revealed that the by-product film thickness is approximately 400 nm (Figs. 2l, m and S14d). The accumulation of many fine grains causes the passivation film to be easily peeled off, which cannot effectively protect the Zn electrode and causes the continuous corrosion of Zn metal. The XPS depth analysis of the O 1*s* orbitals (Fig. 2p) of this electrode in BE reveals the presence of S–O (532.8 eV) and H–O (531.3 eV) at the surface and deep depths without any significant change in intensity. The etch profile of the S 2*p* orbitals (Fig. 2q) indicates the presence of elemental S in the depth direction, which confirms the severe degradation of the Zn electrode caused by Zn_4_(OH)_6_SO_4_·xH_2_O. Different from the hexagonal Zn sheets in the previous reports [60], the present study shows that the deposition topography presents a smaller spherical structure (Figs. 2j and S11). In addition, the significant difference in the proportion of elemental S as well as O between the two different sediments (Fig. S12) suggests the presence of other substances on the surface of the spherical particles on the Zn anodes. The measurement of lattice fringes and SAED both indicates that the SEI film consists of ZnO with a wurtzite structure (P6mc, JCPDS: 36–1451) (Figs. 2k and S13a) [61]. The SEI film thickness was analyzed using FIB-SEM. Based on SEM images and EDS results, the ZnO SEI film thickness was determined to be 31.18 nm (Figs. 2n, o and S14b).

The bond states in the depth direction of the Zn electrode were analyzed using etching XPS of cycled electrode in APM-BE. The O 1*s* spectrum of Zn anode in APM-BE electrode showed the peak split into the adsorption peak of C–O (531.9 eV) and Zn–O (530.0 eV) (Fig. 2r). Both Zn–O and C–O peaks are present with varies of etching depth, indicating that the composition of the *in situ*-formed SEI film is consistent in the bulk structure. The S 2*p* spectrum (Fig. 2s) also shows that the ZnO-based SEI can effectively prevent the formation of Zn_4_(OH)_6_SO_4_·xH_2_O. It is noteworthy that the depth profiling of the C 1*s* orbitals indicates that some APM molecules will be embedded in the process of SEI formation (Fig. S15). Previous studies have consistently shown that the Zn 2*p*_3/2_ binding energy is 1021.7 eV for Zn, while the corresponding peak for ZnO is approximately 1022.0 eV [62]. However, the deep Zn 2*p* orbital peak moves by 1.5 eV toward lower binding energy relative to the surface, which was not found for the electrode circulating in the BE (Fig. S16). It is reasonable to assume that an interfacial effect occurs at the interface between the Zn substrate and the SEI film, resulting in an increase in the electron cloud density of Zn atoms. This leads to a change in the outer electronic structure of the Zn atoms, which accelerates the rate of electron mobility at the interface between the Zn and the ZnO-based SEI. Additionally, this effect strengthens the attraction of the O element in the protective film to the Zn^2+^ in the electrolyte [63].

### Formation Mechanism of ZnO-Based SEI Film

**Fig. 3:**
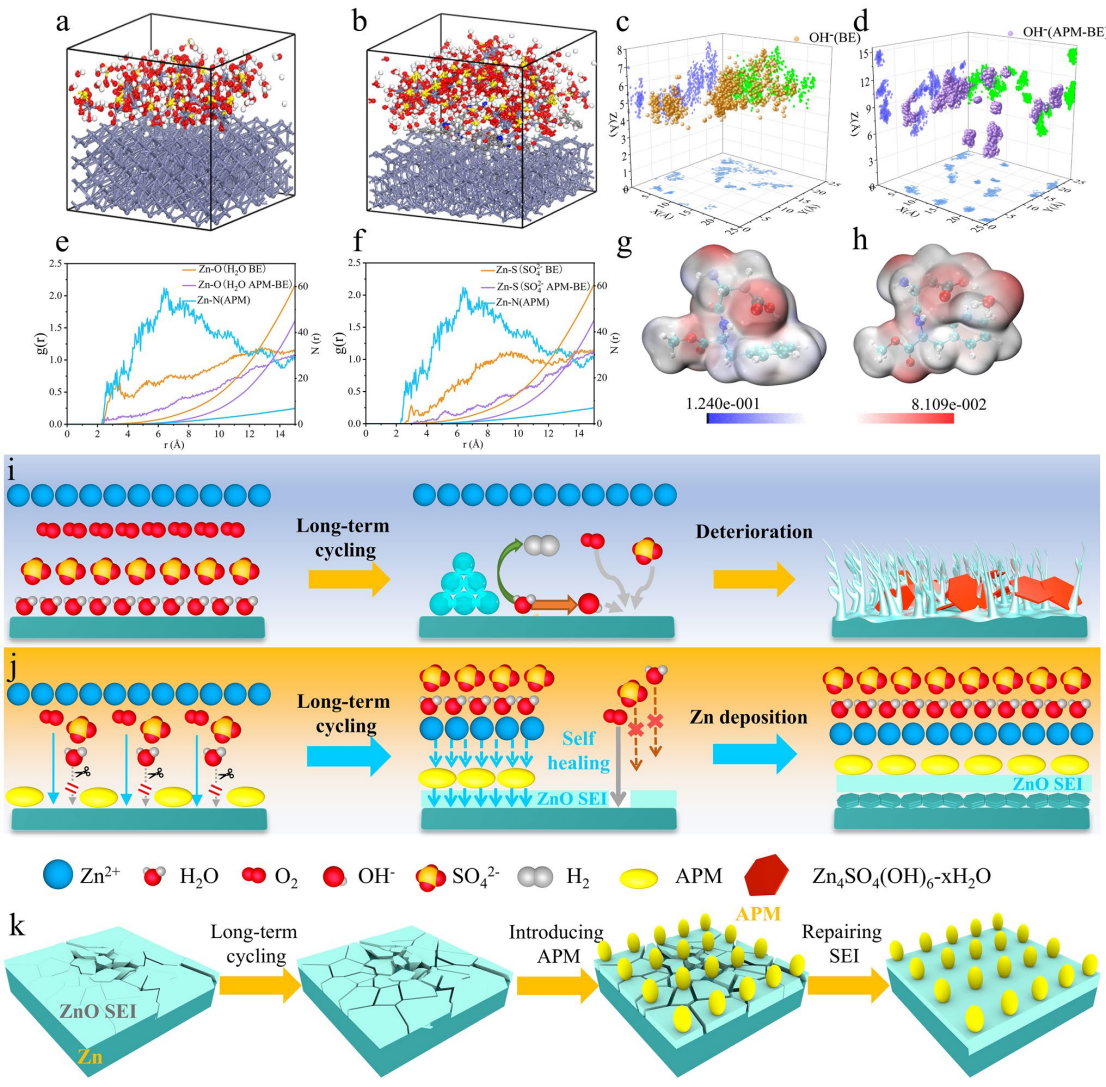
Formation analysis of ZnO-based SEI film. MDS in different systems modeled by **a** BE and **b** APM-BE. Simulation of the center of mass distribution of OH^−^ in different systems (**c** BE and **d** APM-BE). RDF of different systems **e** SO_4_^2−^ and **f** H_2_O. Electron cloud density profiles of **g** APM molecules and **h** APM bound to H_2_O molecules (unit: e bohr^−3^). Schematic illustrations of Zn electrode under electrochemical cycling in **i** BE, **j** APM-BE and **k** the *in situ* repairation of ZnO-based SEI

In order to elucidate the particle distribution characteristics on Zn electrode and the formation mechanism of ZnO-based SEI film, computational models were constructed to simulate the Zn electrode interface in different electrolytes. MD simulations were performed to analysis the spatial arrangement of ionic/molecules species at the electrode/electrolyte interfaces. As depicted in Fig. 3a, b, the comparative configurations were established for BE and APM-BE. The BE model is based on a Zn metal substrate, with H_2_O, SO_4_^2−^, and Zn^2+^ distributed on its surface. In contrast, the APM-BE model additionally incorporates APM molecules. These models are designed to simulate the real electrochemical environment of the Zn electrode in the electrolyte. The centroid distribution of OH^−^ is statistically obtained from the aforementioned models. In BE, the projection of OH^−^ exhibited a concentrated spatial localization within the XY-plane projection with lower distances (average distance: 5.16 Å), suggesting a high propensity for the inhomogeneous formation of Zn_4_(OH)_6_SO_4_·xH_2_O phases on the surface (Fig. 3c). By contrast, APM-BE demonstrated a discrete distribution state, maintaining OH^−^ at substantially with a greater distances away the XY-plane (average distance: 9.40 Å), demonstrating that there is a weak adsorption trend between OH^−^ and Zn electrode under APM-BE system (Fig. 3d). Figure 3e, f illustrates the RDF of H_2_O and SO_4_^2−^ in the electrolyte with respect to the Zn electrode. The particle density of APM molecules near the Zn electrode is significantly higher than that of H_2_O and SO_4_^2−^. Due to the strong adsorption of APM, the diffusion of H_2_O and SO_4_^2−^ to the Zn electrode surface is hindered, leading to a decrease in their concentration at the Zn electrode interface.

Figure 3g, h presents the natural charge distribution of APM molecules and APM-H_2_O complexes, calculated using the Gaussian 16 software package. It can be concluded that all oxygen-containing functional groups in APM molecules, including carboxyl (–COOH), amide (–CONH–), and ester (–COOCH_3_) groups, exhibit a strong negative charge. By virtue of this strong negative electronegativity, the APM molecules embedded in the SEI film accelerate the diffusion rate of positively charged Zn(H_2_O)_6_^2+^ (Fig. S17b) from the electrolyte bulk phase to the surface of the Zn electrode. Therefore, the interfacial APM molecules exhibit dual functionality in accelerating both the mass transport of Zn(H_2_O)_6_^2+^ from bulk electrolyte to Zn electrode interface and subsequent desolvation kinetics [26], which can be further corroborated by Arrhenius analysis of desolvation activation energies, revealing a reduced energy barrier of 54.92 kJ mol^−1^ for APM-BE compared to 61.57 kJ mol^−1^ in BE (Fig. S18). And as the above-mentioned XRD results, the adsorption of APM on the Zn (002) crystalline surface is preferred over the Zn (100) crystalline surface, and this adsorption on a specific crystalline surface induces the deposition of Zn^2+^ on the Zn (002) crystalline surface (Fig. S19). Additionally, the side reactions of the Zn (002) crystal faces are effectively suppressed due to their strong Zn atom trapping ability and high free hydrogen precipitation energy [64]. While the dehydration of Zn(OH)_2_ has been proposed as a plausible pathway for ZnO-based SEI formation on Zn electrode, thermodynamic calculations reveals a positive Gibbs free energy (ΔG = 395.03 kcal mol^−1^) for this reaction (Table S2). This conclusively indicates the hydrogen bonding interactions between APM and H_2_O molecules is insufficient to rupture the chemical bonds within hydroxide species. Consequently, as Zn(OH)_2_ dehydration cannot thermodynamically sustain ZnO-based SEI formation, the oxidation of Zn by dissolved O_2_ emerges as the predominant formation pathway for ZnO. The mechanistic pathway proceeds via the following sequence: the adsorption of APM molecules on the surface of Zn electrode induces the formation of an electrical double layer (EDL) at the Zn/electrolyte interface [58], establishing an electrostatic shielding effect that mitigates Zn electrode degradation by both H_2_O and SO_4_^2−^ species. Although previous study [65] has suggested that dissolved O_2_ typically accelerates the corrosion of Zn, the APM-derived adsorption layer effectively suppressed the parasitic reaction 6Zn + 3O_2_ + (6 + 2x)H_2_O + 2ZnSO_4_ → 2Zn_4_SO_4_(OH)_6_·xH_2_O through steric hindrance and charge repulsion toward interfacial H_2_O and SO_4_^2−^. Consequently, O_2_ was exclusively mediated to participate in the thermodynamically favored formation of ZnO-based SEI through the simplified pathway: Zn + ½O_2_ → ZnO.

Notably, under prolonged cycling conditions, Zn electrodes are susceptible to aqueous corrosion and dendrite growth, eventually leading to electrode failure (Fig. 3i). Although ZnO-based SEI mitigates these deterioration pathways, its structural integrity progressively deteriorates in weakly acidic electrolytes due to corrosive dissolution. Crucially, interfacial APM molecules persistently mitigate H_2_O and SO_4_^2−^ species penetration via steric electronic blocking, while dissolved O_2_ regenerates the SEI through dynamic oxidative repairation (Fig. 3j–k). Distinct from conventional additives that form SEI film on the surface of Zn electrode by self-sacrificial decomposition [66], APM maintains non-consumptive adsorption at the interface, thereby preserving ZnO-based SEI continuity. The electron-donating functional groups (–COOH [67], –NH [68], –OH [69]) can mediate dual interfacial interactions. They can selectively coordinate with Zn(H_2_O)_6_^2+^ to optimize ion desolvation kinetics and electronically couple with ZnO to enhance its stability, which enable simultaneous electrode protection and sustained Zn^2+^ mobility. Collectively, APM additives confer dynamic self-optimization capabilities to Zn surface SEI through synergistic integration of persistent corrosion suppression, oxygen-mediated self-healing functionality, and non-consumptive interfacial stabilization.

### Corrosion and Deposition in Zn Anodes Modulated by APM-Induced SEI

**Fig. 4:**
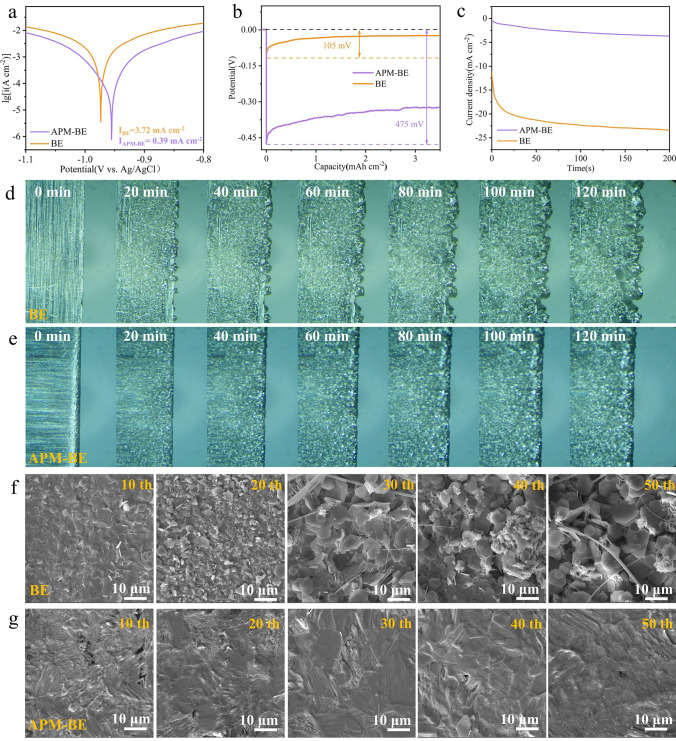
Electrochemical testing of two electrolytes and Zn^2+^ deposition behavior. **a** Tafel tests of different electrolytes in Zn║Zn symmetrical cell. **b** Nucleation overpotentials tests of Zn^2+^ in different electrolytes. **c** Chronoamperometry tests of Zn electrode in different electrolytes. *In situ* optical microscope images of Zn^2+^ deposition in **d** BE and **e** APM-BE at 5 mA cm^−2^–5 mAh cm^−2^. *Ex situ* SEM images of a Zn electrode in **f** BE and **g** APM-BE at 1 mA cm^−2^–1 mAh cm^−2^

The construction of a ZnO-based self-healing SEI fundamentally alters the physicochemical properties of Zn electrodes, particularly impacting the corrosion resistance in aqueous electrolyte systems. Given the well-established thermodynamic instability of Zn in aqueous environments, where spontaneous corrosion occurs through H_2_O induced degradation pathways [70], the protective mechanism of ZnO-based SEI film on Zn electrodes was systematically investigated. Electrochemical polarization measurements reveal distinct corrosion behavior modification (Fig. 4a). The Zn electrode exhibits a corrosion potential of -0.974 V with a corresponding current density of 3.72 mA cm^−2^. In contrast, Zn electrode in APM-BE demonstrates an improved corrosion resistance, as evidenced by a 21 mV positive shift in corrosion potential (− 0.953 V) and a remarkable 89.5% reduction in corrosion current density (0.39 mA cm^−2^). This protective effect is further corroborated by the LSV analysis (Fig. S20), where the APM-BE system requires a significantly lower overpotential (− 1.17 V vs. Ag/AgCl) compared to BE system (− 1.11 V vs. Ag/AgCl) to achieve a current density of 25 mA cm^−2^. Furthermore, to evaluate long-term stability, we conducted post-immersion characterization of Zn electrodes’ morphology and composition. Surface analyses via SEM and XRD reveal that Zn foils immersed in APM-BE exhibit reduced and flattened Zn_4_(OH)_6_SO_4_·xH_2_O deposits. Moreover, Zn foils treated with APM-BE can retain electrochemical cyclability in Zn║Zn symmetrical batteries even after prolonged immersion (Fig. S21). These findings confirm that the APM-induced SEI film effectively mitigates Zn electrode corrosion.

Apart from the corrosion of Zn metal electrode in aqueous electrolyte, dendrite growth stemming from uneven Zn deposition is another critical issue compromises the safety and cyclability of AZIBs. Therefore, systematic investigation of Zn^2+^ electrodeposition dynamics is necessary. The significant decrease in response current observed in the CV curves together with the slight increase in interfacial transfer impedance in EIS test (Fig. S22) indicates that the APM-induced SEI increases the potential barrier for Zn^2+^ transfer to the Zn metal surface. As shown in Fig. 4b, the nucleation overpotential of the Zn electrode in BE and APM-BE is 105 and 475 mV, respectively. According to previous studies on Zn electrode deposition, a decreased nucleation overpotential leads to fewer and more sparsely distributed nucleation sites, thereby promoting the growth of Zn dendrites [71]. On the contrary, at higher nucleation overpotentials, a larger number of smaller and uniformly distributed initial Zn nuclei are favorable for guiding the subsequent deposition of Zn, guaranteeing a smooth surface without dendrites [72]. The chronoamperometric (CA) current curves in Fig. 4c demonstrate that the current density of the Zn electrode in BE exhibits a continuous decline. This suggests that Zn^2+^ undergoes prolonged 2D diffusion on the electrode surface. Their continuous deposition results in the formation of Zn dendrites at only a limited number of active sites, ultimately causing cell failure. In contrast, the current density of the Zn electrode in APM-BE remains stable, suggesting a brief 2D diffusion followed by compact 3D diffusion at the SEI interface. In summary, the ZnO-based SEI film effectively significantly inhibits the aqueous electrolyte corrosion and the formation of Zn dendrites. Additionally, the surface-adsorbed APM lowers the desolvation energy of hydrated Zn^2+^. These two factors combined significantly prolong the lifespan of Zn electrode.

In situ optical microscopy validates the dendrite suppression mechanism of APM-induced SEI through real-time monitoring of Zn deposition dynamics. Figure 4d and Video S1 illustrate that the Zn electrode in BE exhibits uneven crystal nuclei after just 20 min of reaction. As deposition continues, Zn^2+^ deposits onto these few crystal nuclei, leading to the formation of Zn dendrites. In contrast, Fig. 4e and Video S2 display a dense Zn deposition, resulting in a flat and dendrite-free surface during the continuous electrode position process. By maintaining a constant current density and continuously extending the cycling time, SEM reveals that the electrode surface in APM-BE exhibits a flat and dense deposition film due to the formation of a ZnO-based self-healing SEI. Upon cycling, the film gradually closes, and no localized accumulation of growing Zn dendrites is observed. In contrast, the Zn electrode in BE shows surface deterioration and the formation of dendritic structures due to the disordered deposition of hexagonal Zn platelets and the continuous generation of Zn_4_(OH)_6_SO_4_·xH_2_O (Fig. 4f, g). Furthermore, the digital images reveal that APM-BE produces clean septa and flat Zn electrode even after 100 h (Fig. S23). Previous study using DFT calculations has demonstrated the strong affinity of ZnO for Zn^2+^ and the accelerated migration of Zn^2+^ within the ZnO lattice [61, 62]. Different capacities exhibited by coin cells assembled with ZnO and Zn foil using ZnSO_4_·H_2_SO_4_ electrolytes of the same pH confirm the Zn^2^⁺ storage capability of ZnO (Fig. S24).

**Fig. 5:**
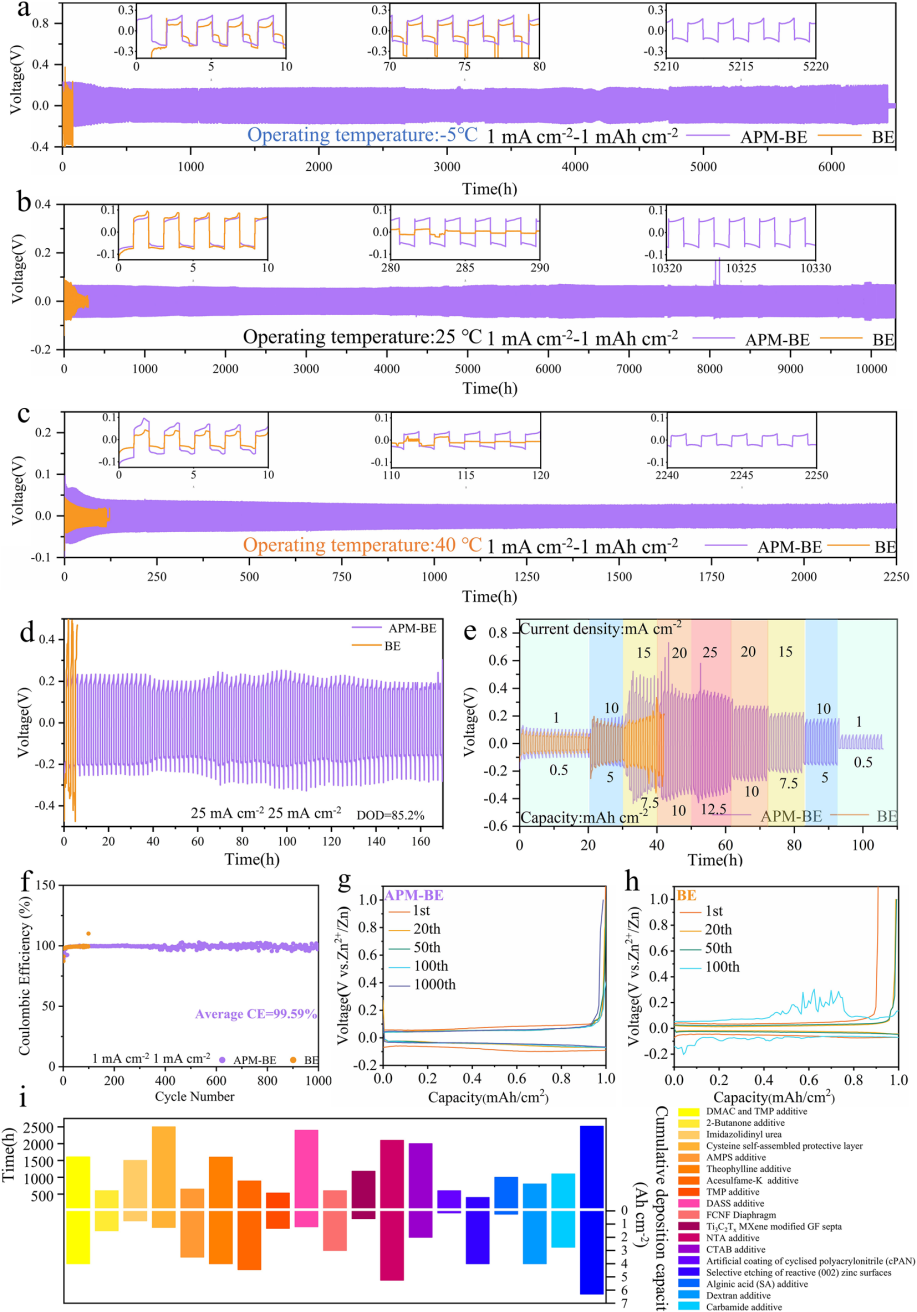
Electrochemical performance of Zn║Zn symmetrical cells. **a–c** Cycle test of Zn║Zn symmetric cells at different test temperatures at 1 mA cm^−2^–1 mAh cm^−2^. **d** 25 mA cm^−2^–25 mAh cm^−2^ Zn║Zn symmetric cells cycle. **e** Multiplication performance test of Zn║Zn symmetric cells. **f** Coulombic efficiency of Zn║Cu half-cells at 1 mA cm^−2^–1 mAh cm^−2^. Voltage profiles of Zn║Cu half-cells in different electrolytes **g** APM-BE and **h** BE. **i** Comparison of in-cycle life and cumulative deposition capacity of APM additives with other published works

The application of AZIBs in both high- and low-temperature environments is crucial for their commercialization. However, it is widely recognized that drastic temperature changes can significantly impact the performance of aqueous batteries [73]. Higher temperatures accelerate the corrosion of Zn electrode by H_2_O molecules, leading to more rampant growth of Zn dendrites, which significantly shortens the lifespan of the cell [74]. Lower temperatures can significantly reduce the ionic conductivity of the electrolyte and even cause it to freeze, resulting in severe volume expansion [75]. Previous studies often required the introduction of large amounts of organic solvents to mitigate the harsh effects of H_2_O molecules. However, the use of organic solvents reduces the conductivity of the electrolyte, slows down the deposition kinetics of Zn^2+^, and compromises the safety and environmental benefits of aqueous batteries [76]. DSC testing indicates that APM-BE has a wider thermal stability range than BE, with a freezing point of − 11.62 °C and a boiling point of 110.73 °C (Fig. S25). Attributed to the protecting of ZnO-based SEI on Zn electrode, under 1 mA cm^−2^–1 mAh cm^−2^, Zn║Zn symmetric cells can run for more than 6400, 10,330, and 2250 h, respectively, at − 5, 25, and 40 °C. Moreover, it can still run stably under higher test conditions (Figs. 5a–c and S26–S28). In addition, APM additives have a wide range of compatibility; with the introduction of APM into halide salts (ZnBr_2_) and organic salts (Zn(OTF)_2_), the cycle life reached 1300 and 980 h, respectively (Fig. S29).

On the other hand, the energy density of AZIBs is also influenced by the adaptability of Zn electrode to varying depth of discharge (DOD). However, at very high discharge depths, the inhomogeneity of the surface of the Zn electrode can cause the electrode to fail due to the gradual stripping of Zn^2+^ and the creation of voids in the electrode. Under the test conditions of 25 mA cm^−2^–25 mAh cm^−2^ (Fig. 5d), the DOD has reached 85.2%, while the Zn║Zn symmetrical cell using APM-BE can still run stably for more than 160 h, and the ZnO-based SEI film remains stable (Fig. S30). Comparison of APM additives with other strategies for stabilizing Zn electrode is shown in Fig. 5i and Table S3. The accumulated deposition capacity of Zn electrode stabilized by introducing APM into the electrolyte at different discharge depths is relatively high in the current studies of various stabilized Zn electrode. In the multiplication test, the Zn║Zn symmetrical cells using APM-BE can remain stable from 0.5 to 12.5 mAh cm^−2^ changes and its voltage returned to normal after the current returned to its initial value (Fig. 5e).

The stability of the Zn electrode in the long cycle was characterized by testing the CE of the Zn║Cu half-cells with the Cu electrode as the counterelectrode. As shown in Fig. 5f, the cells with APM-BE remain stable throughout the test for up to 1,000 cycles, maintaining an average CE as high as 99.59%. The stability is further confirmed by the highly overlapping voltage profiles during the selected cycles, as illustrated in Fig. 5g. The voltage of the cell with BE fluctuates abnormally after fewer than 100 cycles (Fig. 5h), indicating that the cell fails at this point. Digital photographs and SEM images of Cu electrode after cycling in different electrolyte systems demonstrate that Zn deposition is more uniform with APM-BE (Fig. S31a–c). In contrast, with BE, Zn^2+^ deposits occur only in localized areas and the continuous growth of Zn dendrites ultimately leads to cell failure (Fig. S31d–f). The addition of APM results in a significant increase in the polarization voltage of the cell, rising from 80.2 to 120.4 mV. This suggests that APM reduces the size of the Zn^2+^ nucleation sites, promoting a more homogeneous deposition film (Fig. S32). Even under the more demanding conditions, the average CE is still close to 100% (Fig. S33). These results demonstrate that the synergistic effect of the self-healing ZnO-based SEI and the embedded APM molecules significantly enhances the electrochemical performance of Zn electrode.

### Self-Healing ZnO-Based SEI-Enabled High-Performance Zn‖NH_4_^+^-V_2_O_5_ Full Cells

**Fig. 6:**
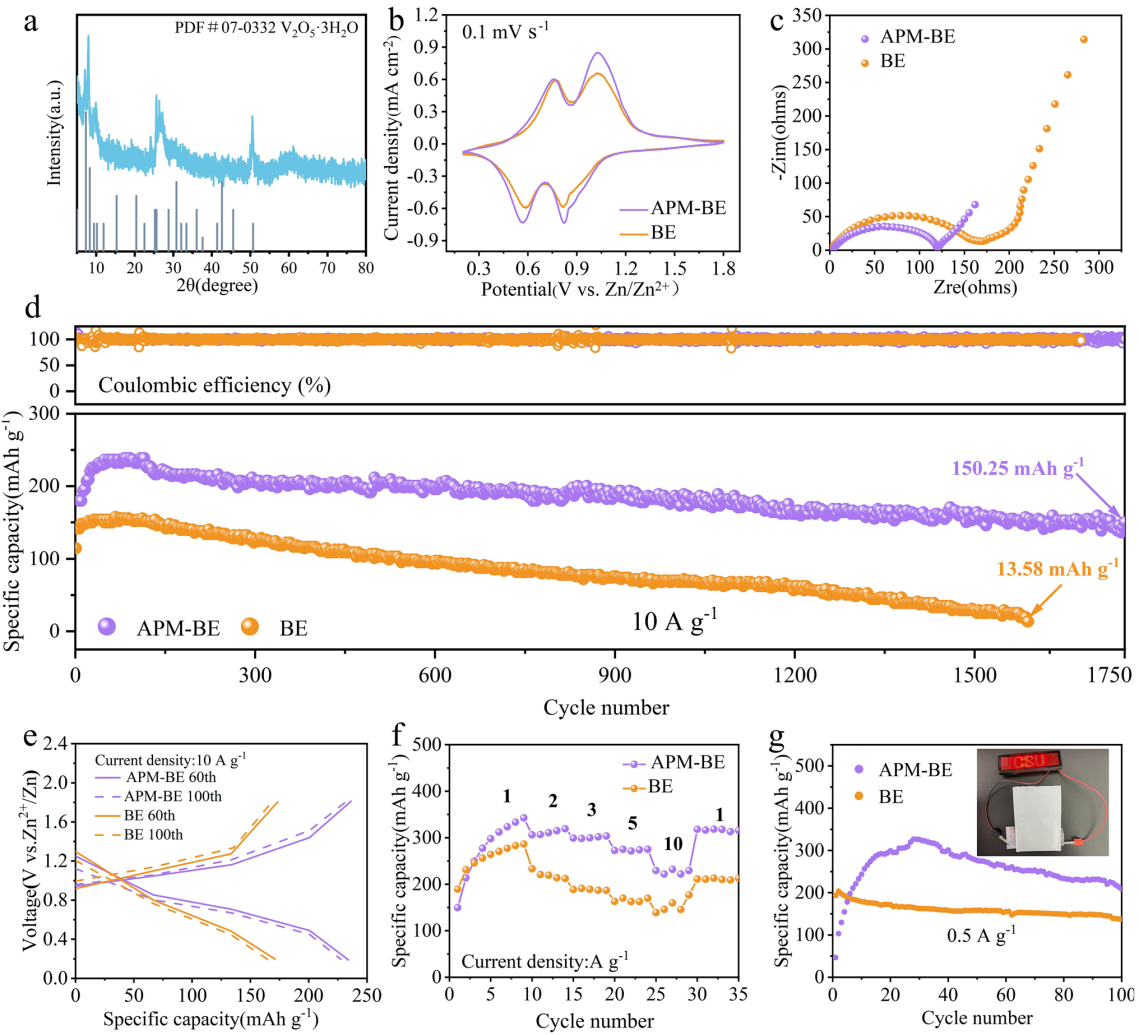
Electrochemical performance and characterization of Zn║NH_4_^+^-V_2_O_5_ full cells. **a** XRD patterns of NH_4_^+^-V_2_O_5_ cathode materials. **b** CV and **c** EIS curves of full cells using different electrolytes at a scan rate of 0.1 mv s^−1^. **d** Cycling test of full cells at a current density of 10 A g^−1^ and **e** voltage curves of selected cycles. **f** Multiplication performance test of full cells. **g** Cycling test of assembled pouch cell at 0.5 A g^−1^

Full cells are assembled using the commonly used vanadium-based (NH_4_^+^-V_2_O_5_) material as the cathode, together with APM-BE and other components, to test the utility of APM additives in AZIBs. Figure 6a shows the XRD of NH_4_^+^-V_2_O_5_ prepared by thermal cracking, with its diffraction peaks corresponding to the PDF card reported in the literature (PDF #07-0332) [77]. The morphology of the cathode material, depicted in Fig. S34a, b, consists of irregularly shaped masses measuring in micrometers, which is consistent with relevant reports. Additionally, the presence of the three elements N, V, and O is clearly visible in the EDS scan (Fig. S34c). Figure 6b shows the CV curves of the full cell in both electrolyte systems, and both systems show characteristic redox peaks at the cathode of V_2_O_5_, which are attributed to the co-embedding mechanism of Zn^2+^ and H^+^, in agreement with the results of the previous studies [78]. It is worthy to note that the affinity for Zn^2+^ of the ZnO-based SEI film is excellent, and the addition of APM increases the current density at the reaction potential and enhances the capacity of the electrochemical system. Furthermore, as demonstrated in Fig. 6c, the inclusion of APM results in a noteworthy decrease in the cell's charge transfer impedance, promoting rapid reaction kinetics. Charge/discharge tests were conducted at a high current density of 10 A g^−1^ (Fig. 6d). The results indicate that the capacity of the batteries using BE decayed severely during cycling and failed at around 1600 turns, with a capacity retention rate of only 10.2%. The full battery using APM-BE exhibits high capacity and exceptional stability even under wide-temperature ranges and low N/P test conditions (Figs. S35, S36). The degradation of the control cell was primarily attributed to corrosion cavities in the Zn electrode and short circuits caused by the growth of Zn dendrites that pierced the diaphragm (Fig. S37a, b). In contrast, the Zn electrode in the full cell using APM-BE is much flatter (Fig. S37c, d). Due to the fast migration rate of Zn^2+^, the discharge capacity can still reach 150 mAh g^−1^ after 1750 cycles, with a capacity retention rate of 77.8%, and the polarization voltage of the full cell was reduced (Fig. 6e). The full cell by using APM-BE exhibits exceptional rate capability, delivering specific capacities of 342.6, 319.1, 303.5, 275.2, and 232.0 mAh g^−1^ at progressively intensified current densities of 1 to 10 A g^−1^, while maintaining 98.2% capacity recovery upon reverting to 1 A g^−1^ after high-rate cycling (Fig. 6f). This remarkable reversibility stems from the self-healing SEI’s dual functionality in suppressing side reactions and dynamically homogenizing Zn^2+^ flux to mitigate polarization-induced capacity degradation. In contrast, full cells using BE suffer obvious capacity decay accompanied by irreversible Coulombic efficiency, a direct consequence of unstable Zn anode. The electrochemical efficacy of APM additives was validated through galvanostatic cycling of pouch cells (Fig. 6g). Cells employing APM-BE exhibited a specific capacity of 326 mAh g^−1^, significantly surpassing BE-based counterparts (200 mAh g^−1^), thereby highlighting the critical role of APM in enhancing Zn deposition reversibility.

## Conclusions

This work presents an O_2_-driven interfacial engineering strategy that constructs dynamically ZnO-based self-healing SEI film to simultaneously mitigate Zn metal anode corrosion and dendrite growth in AZIBs. By precisely regulating APM concentration, we establish a dual protection mechanism where molecular shielding effectively passivates H_2_O/SO_4_^2−^ corrosion while dissolved O_2_ orchestrates in situ SEI formation with continuous ZnO regeneration. The strong shielding of APM against H_2_O molecules and SO_4_^2−^ allows the protection of Zn electrode during the cycles and guarantees the self-healing of ZnO-based SEI film and real-time protection of Zn anodes. Attributed to the protective effect of ZnO-based SEI and the attraction of APM molecules to hydrated Zn^2+^, the long cycling, wide-temperature operation, and high depth of discharge performance of Zn║Zn symmetric cell are achieved. Zn║Zn symmetric cell tests show that it can be cycled at 1 mA cm^−2^ –1 mAh cm^−2^ for more than 10,330 h at ambient temperature and still operate stably at 25 mA cm^−2^–25 mAh cm^−2^ (DOD: 85.2%), up to 6,400 h at low temperatures (− 5 °C), and even more than 2,200 h at high temperatures (40 °C). The assembled Zn║NH_4_^+^-V_2_O_5_ full battery achieves more than 1,700 cycles at a current density of 10 A g^−1^ and still has a capacity of 150.25 mAh g^−1^ capacity, demonstrating the effective protection of ZnO-based SEI on the Zn electrode and the fast conduction of Zn^2+^. This strategy of employing APM as electrolyte additive for the construction of self-healing SEI lay on Zn anodes presents a promising pathway for developing AZIBs capable of operating within wide temperature range, high capacities, long cycle life, and depth-of-discharge conditions, thereby bridging fundamental interface science with practical energy storage application of AZIB.

## Supplementary Information

Below is the link to the electronic supplementary material.

Supplementary file1 (MP4 5609 KB)

Supplementary file2 (MP4 3774 KB)

Supplementary file3 (DOCX 15475 KB)
