# Supplementary material for: Aspartame Endowed ZnO-Based Self-Healing Solid Electrolyte Interface Film for Long-Cycling and Wide-Temperature Aqueous Zn-Ion Batteries
Source: Nanomicro Lett. 2025 May 12;17:254. doi: 10.1007/s40820-025-01765-6 (PMC12069790; doi:10.1007/s40820-025-01765-6)
Supplement: Supplementary file 3 — Supplementary file3 (DOCX 15475 KB) [file 40820_2025_1765_MOESM3_ESM.docx]

Supporting Information for

**Aspartame Endowed ZnO-Based Self-Healing Solid Electrolyte Interface Film for Long-Cycling and Wide-Temperature Aqueous Zn-Ion Batteries**

Yunyu Shi^1^, Yingkang Liu^1^, Ruirui Chang^1^, Guilin Zhang^1^, Yuqing Rang^1^, Zheng-Long Xu^2^, Qi Meng^2^, Penghui Cao^3^, Xiangyang Zhou^1^, Jingjing Tang^1^*, Juan Yang^1^*

^1^ School of Metallurgy and Environment, Central South University, Changsha 410083, P. R. China

^2^ Research Institute for Advanced Manufacturing, Department of Industrial and Systems Engineering, The Hong Kong Polytechnic University, Hung Hom, Hong Kong SAR, P. R. China

^3^ College of Energy and Power Engineering, Changsha University of Science & Technology, Changsha 410114, P. R. China

*Corresponding authors. E-mail: [tangjj@csu.edu.cn](mailto:tangjj@csu.edu.cn) (Jingjing Tang); [j-yang@csu.edu.cn](mailto:j-yang@csu.edu.cn) (Juan Yang)

**Supplementary Figures**


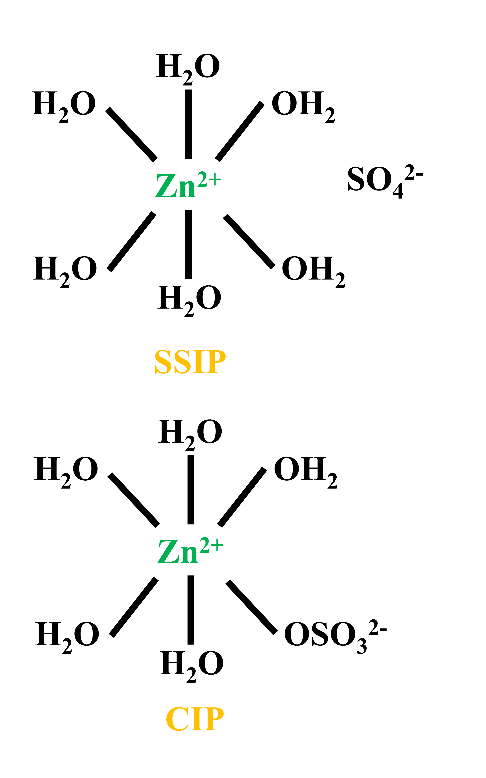


Fig. S1 Schematic diagram of SSIP (Solvent-Separated Ion Pair) and CIP (Contact Ion Pair)


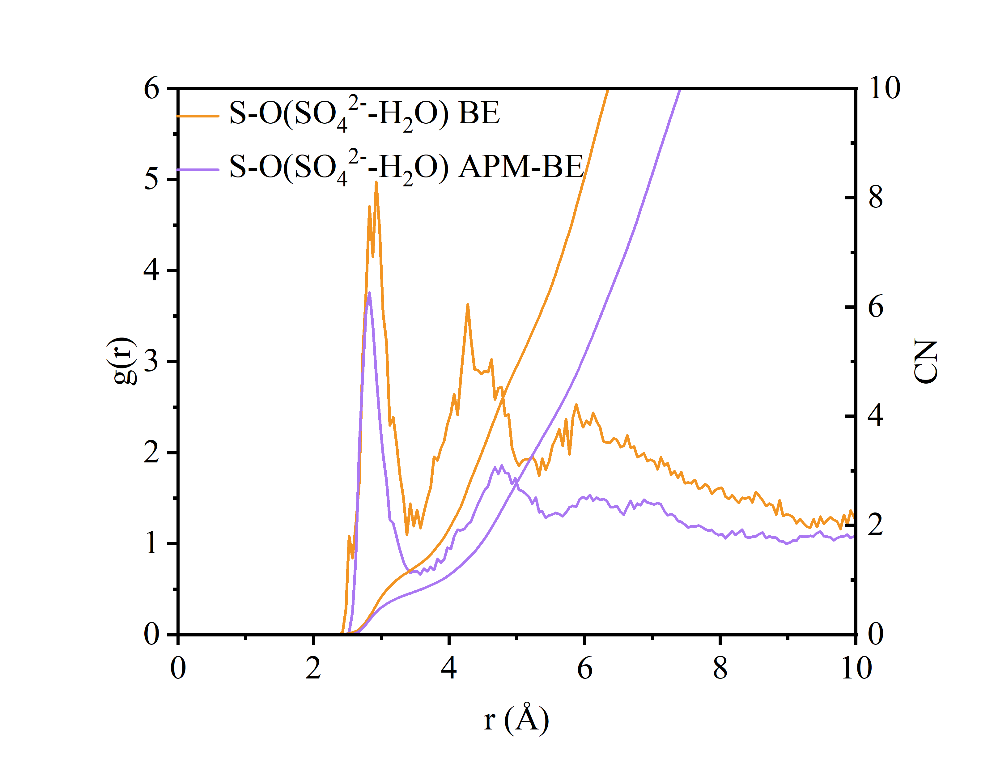


Fig. S2 Radial distribution function(RDF) of SO_4_^2-^ and H_2_O molecules in different electrolyte systems
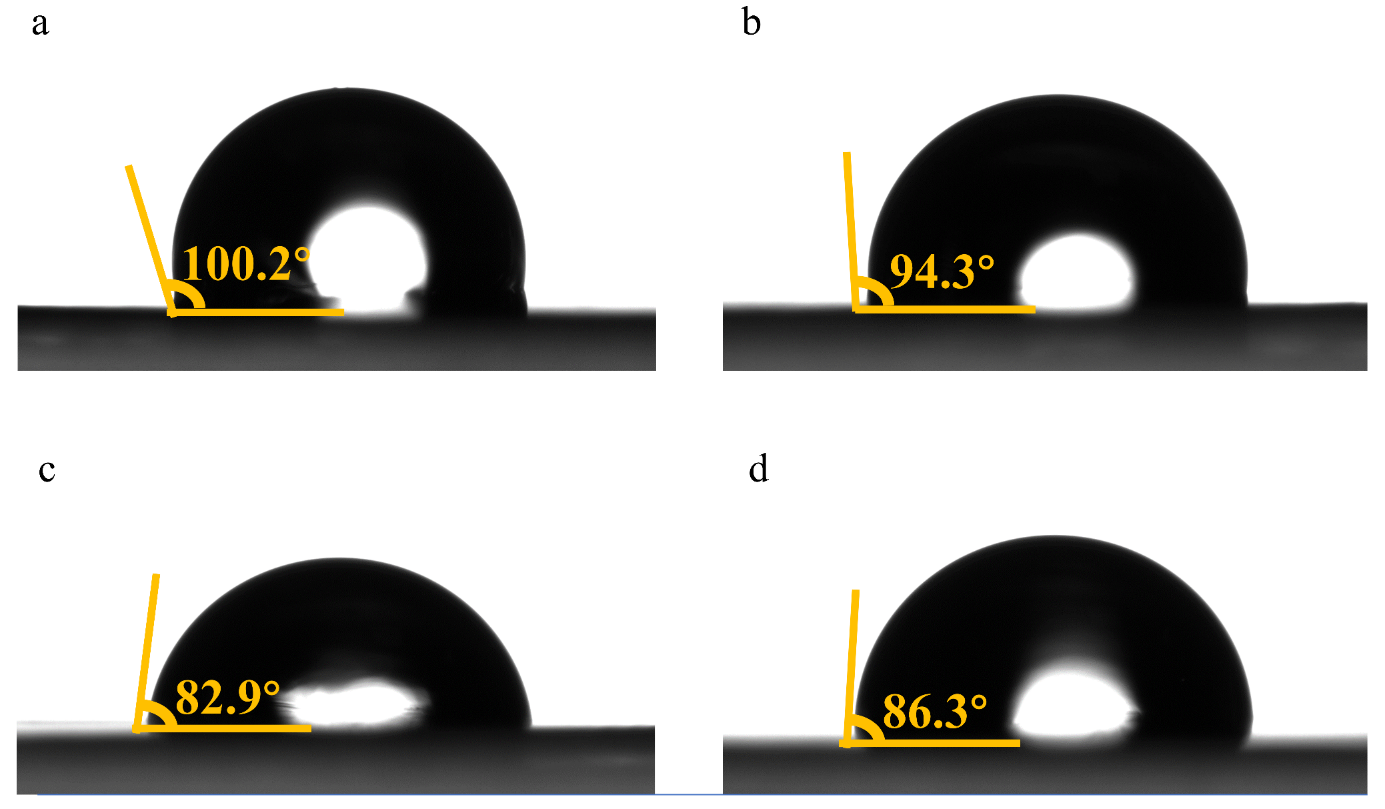


Fig. S3 (a) 0 (b) 0.5 (c) 1 (d) 8 mg/ml contact angle of electrolyte of APM concentration on zinc electrode


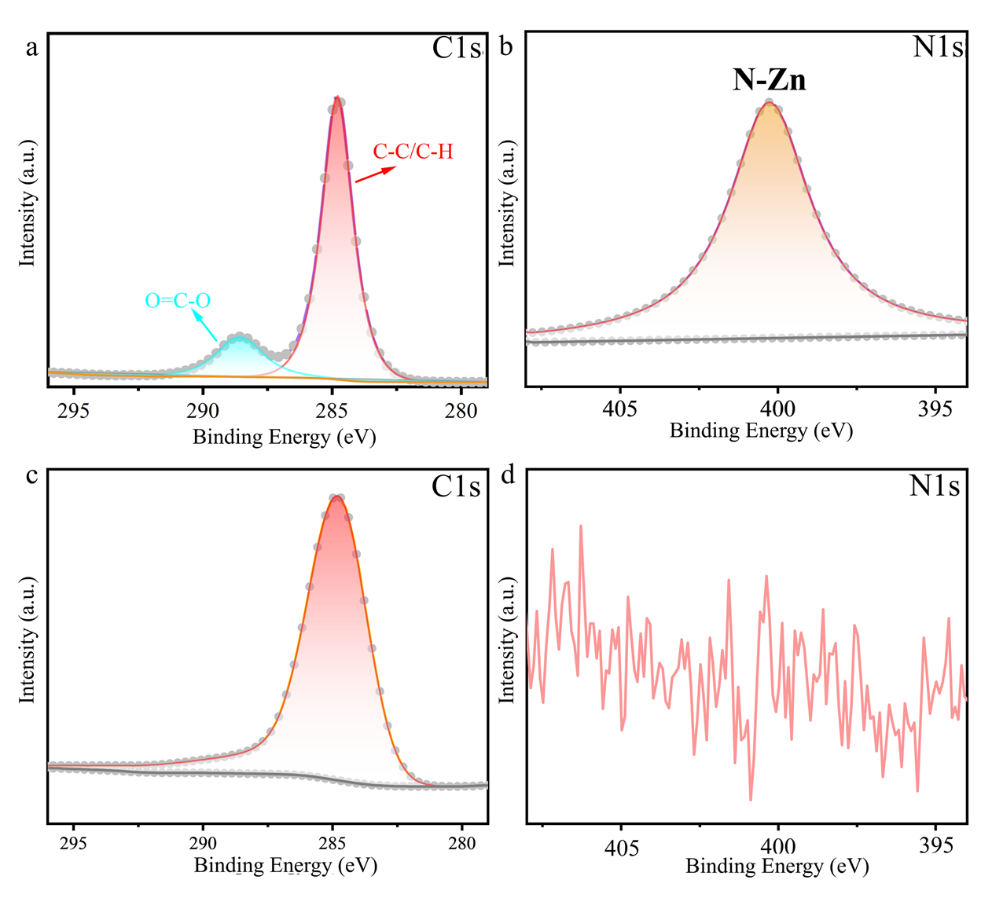


Fig. S4 XPS patterns of Zn electrodes (a-b) using APM-BE and (c-d) using BE after cycling a Zn║Zn symmetric cells for 50 cycle at 5 mA cm^-2^-5 mAh cm^-2^


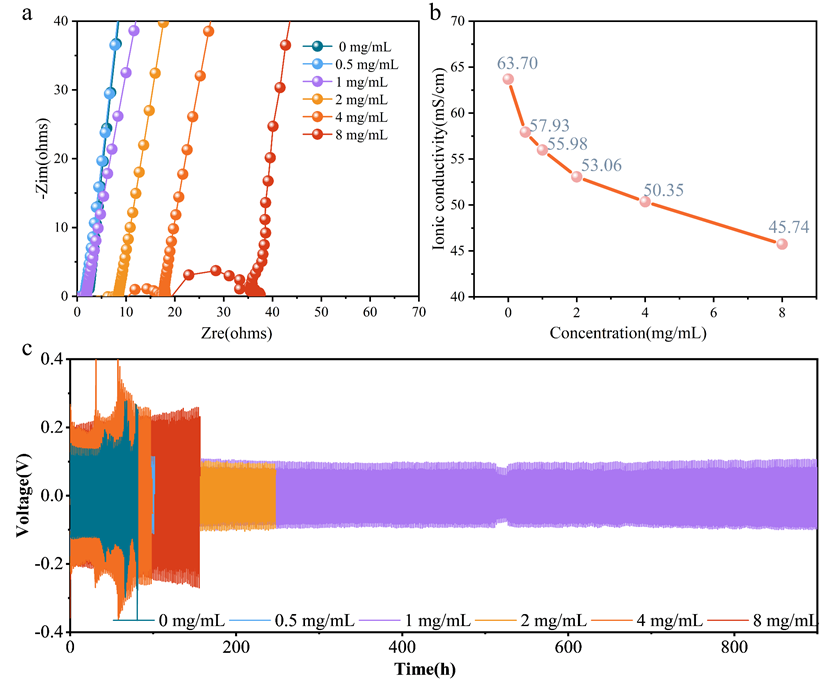


**Fig. S5** (**a**) EIS testing of SS‖SS symmetric cells with different types of electrolytes. (**b**) Plot of conductivity as a function of APM concentration. (**c**) Cycle diagram of Zn‖Zn symmetric cells with different electrolytes under 5 mA cm^-2^-5 mAh cm^-2^


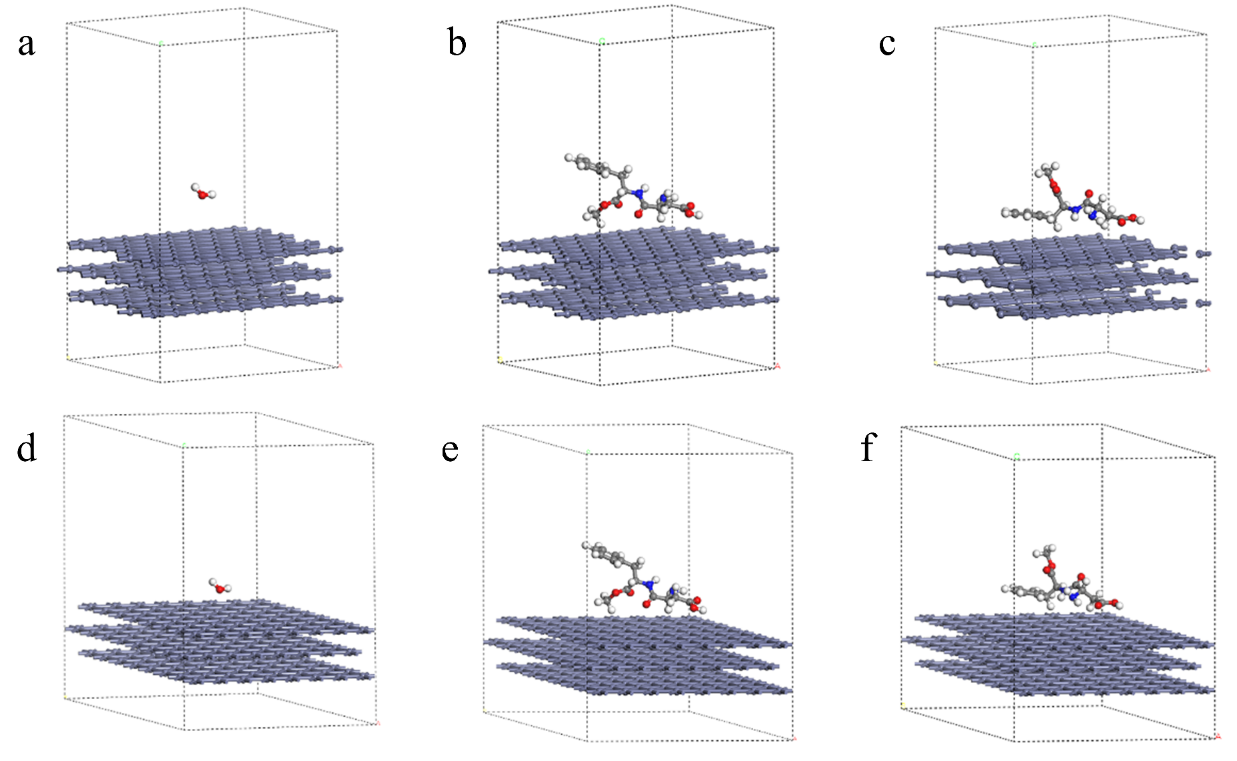


Fig. S6 Slabs for (a) Zn100-H_2_O; (b) Zn100-Apartame-1; (c) Zn100-Apartame-2; (d) Zn002-H_2_O; (e) Zn002-Apartame-1 and (f) Zn002-Apartame-2


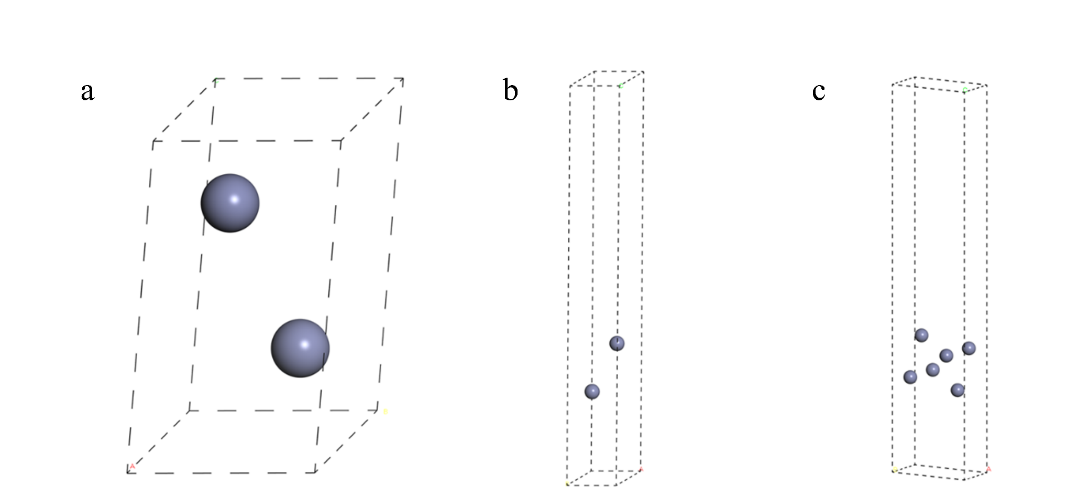


Fig. S7 (a) Cell structure of Zn; slabs of (b) Zn(100) and Zn(002)


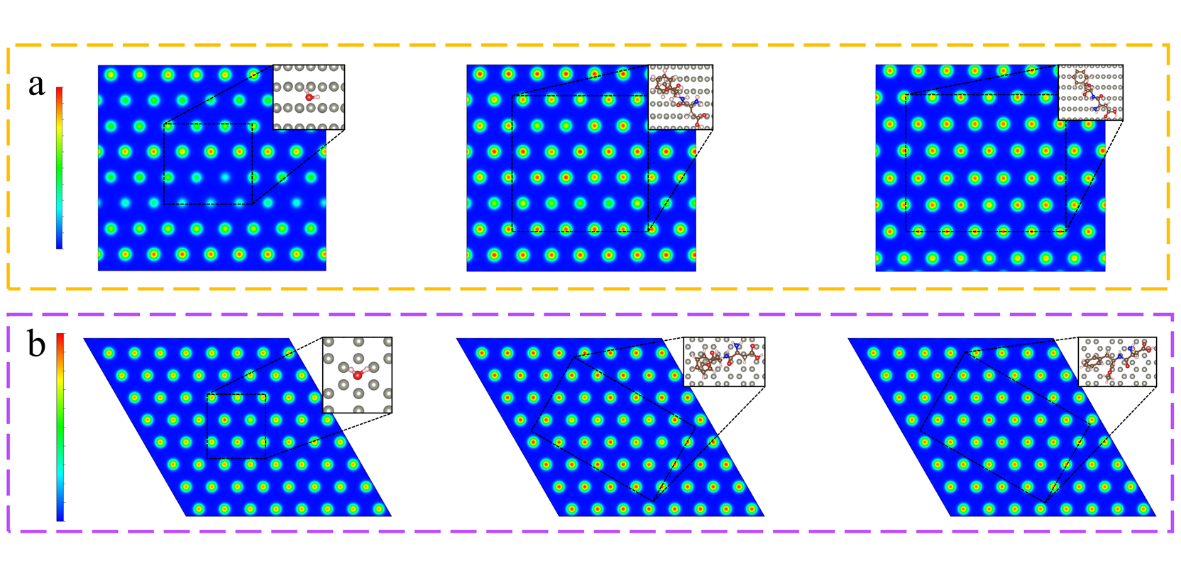


Fig. S8 2D charge density distribution of H_2_O, APM-1 and APM-2 adsorption on g Zn (100) crystal face and h Zn (002) crystal face


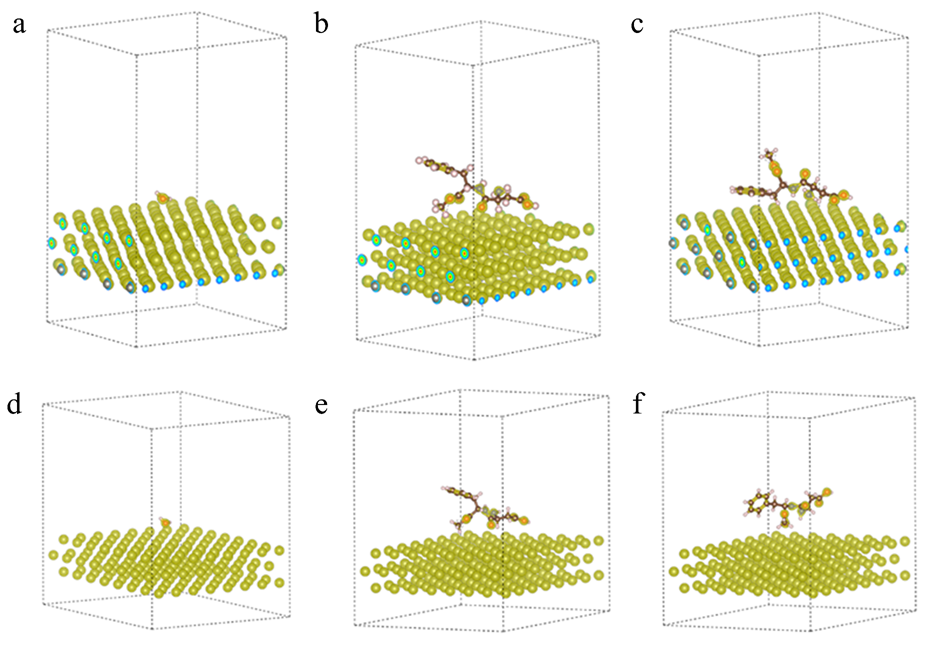


Fig. S9 Charge density distribution of adsorption corresponding Zn surface: (a) Zn100-H_2_O; (b) Zn100-Apartame-1; (c) Zn100-Apartame-2; (d) Zn002-H_2_O; (e) Zn002-Apartame-1 and (f) Zn002-Apartame-2 (Isosurface level :0.27)


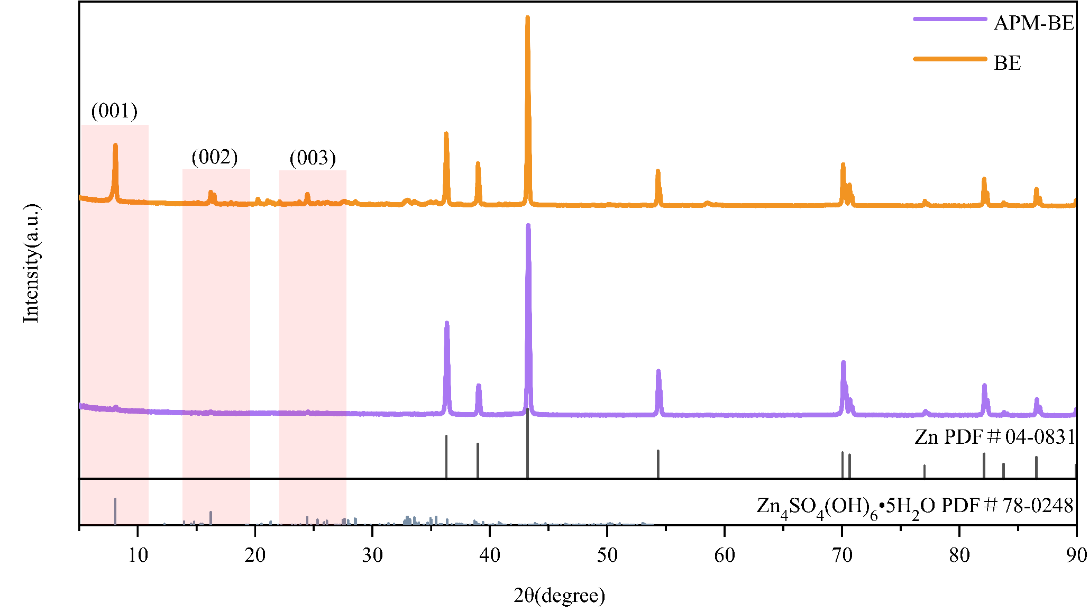


Fig. S10 XRD patterns of Zn electrodes with different electrolytes after 50 cycles at 5 mA cm^-2^ 5 mAh cm^-2^ conditions


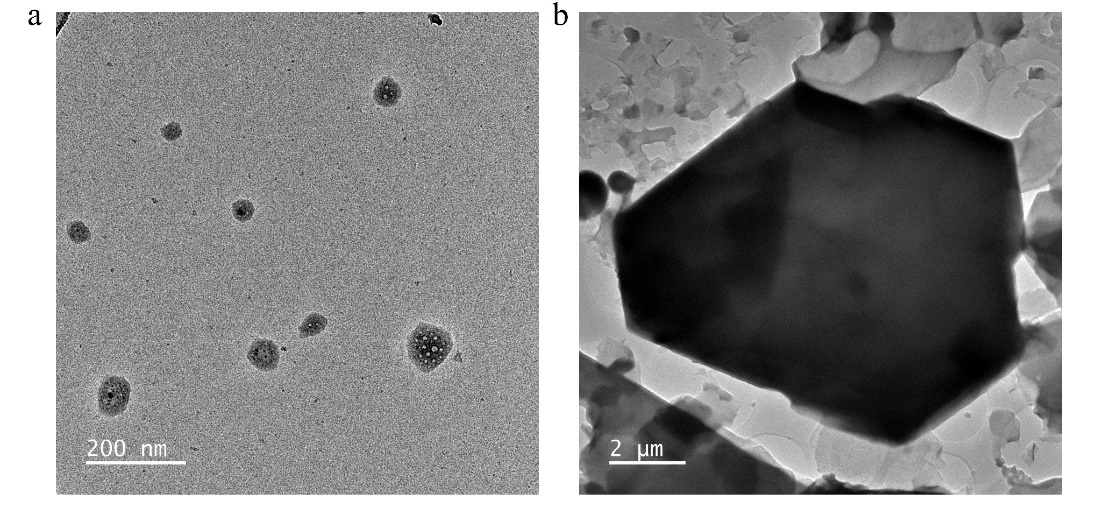


Fig. S11 Deposits on the surface of the Zn electrode corresponding to the (a) APM-BE (b) BE electrolyte after 50 cycle of cycling at 5 mA cm^-2^-5 mAh cm^-2^


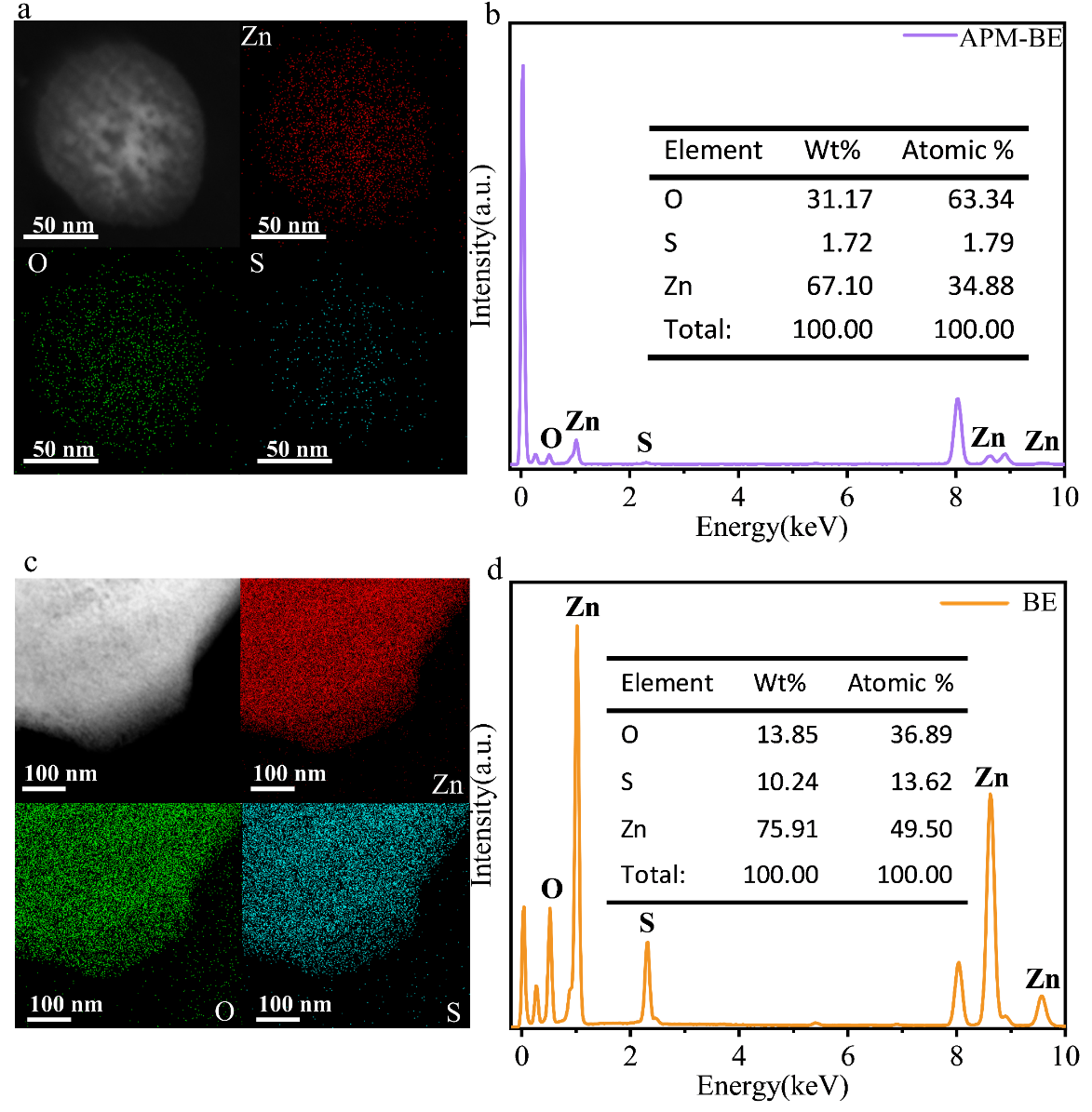


Fig. S12 EDS mapping of deposits corresponding to (a) APM-BE and (c) BE. Elemental content of deposits corresponding to (b) APM-BE and (d) BE


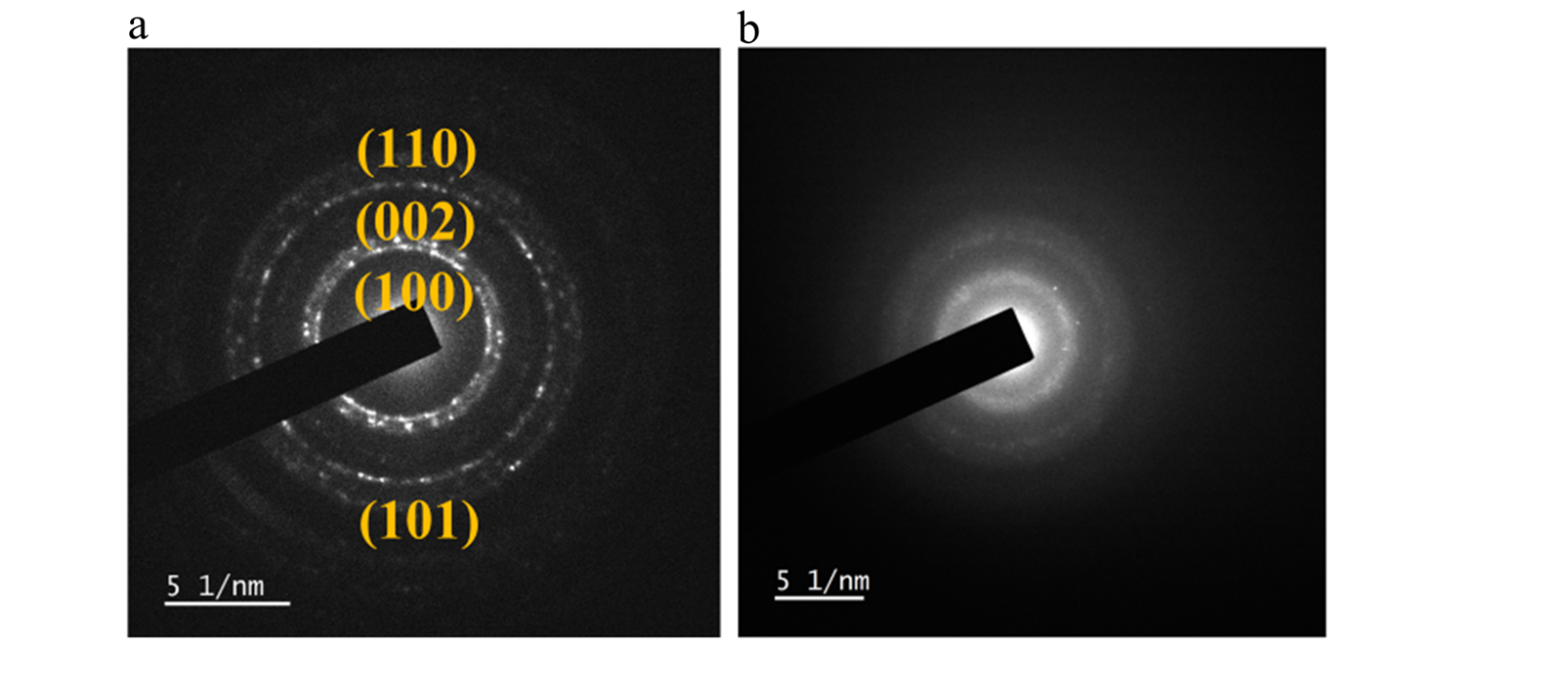


Fig. S13 Selected Area Electron Diffraction (SAED) Images of Different Samples


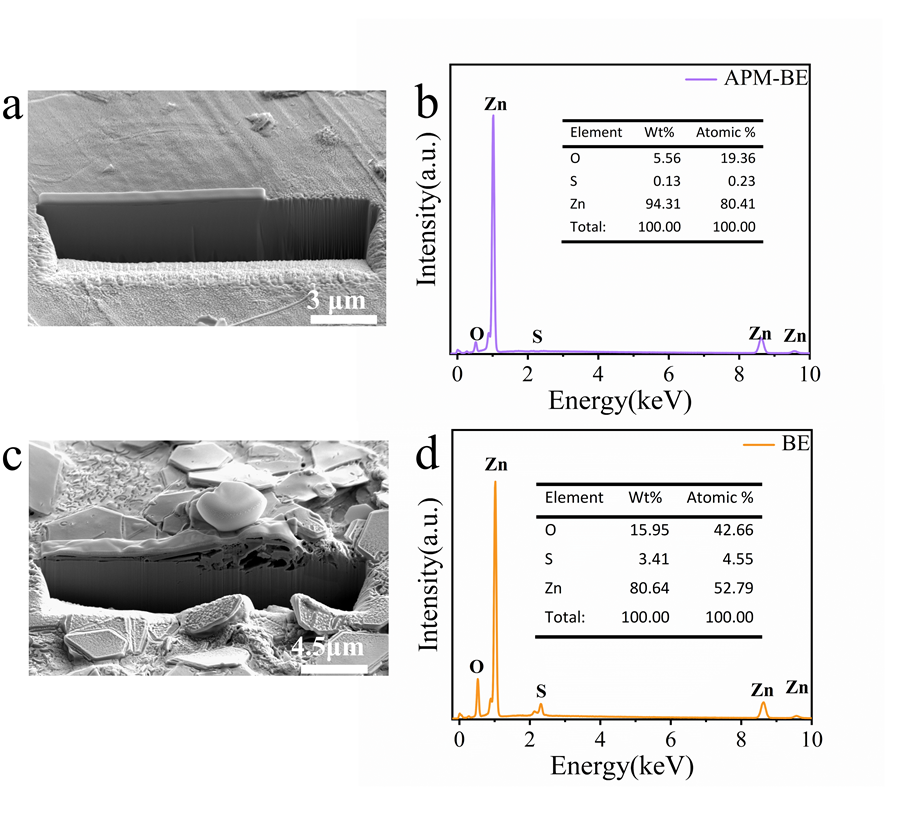


Fig. S14 (a) SEM image after 50 cycles in APM-BE and (b) corresponding elemental mapping from Fig. 2i. (c) SEM image after 50 cycles in BE and (d) corresponding elemental mapping from Fig. 2p


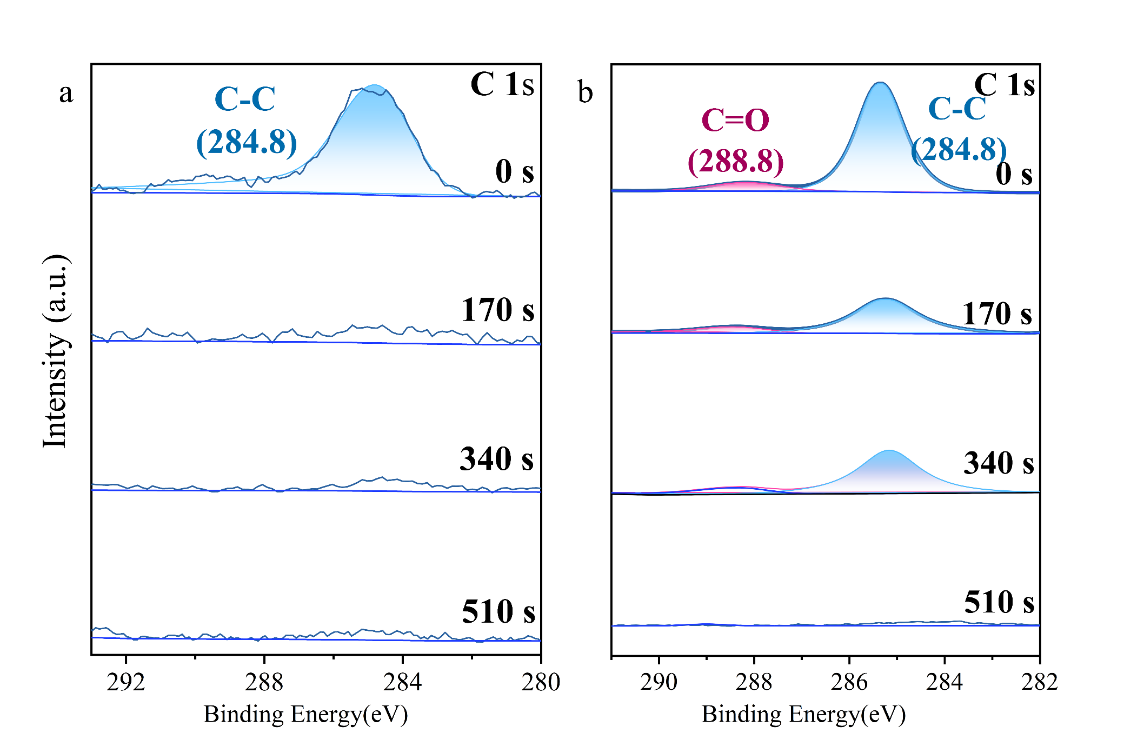


Fig. S15 XPS depth profiles of Zn electrode C1s (a) BE and (b) APM-BE


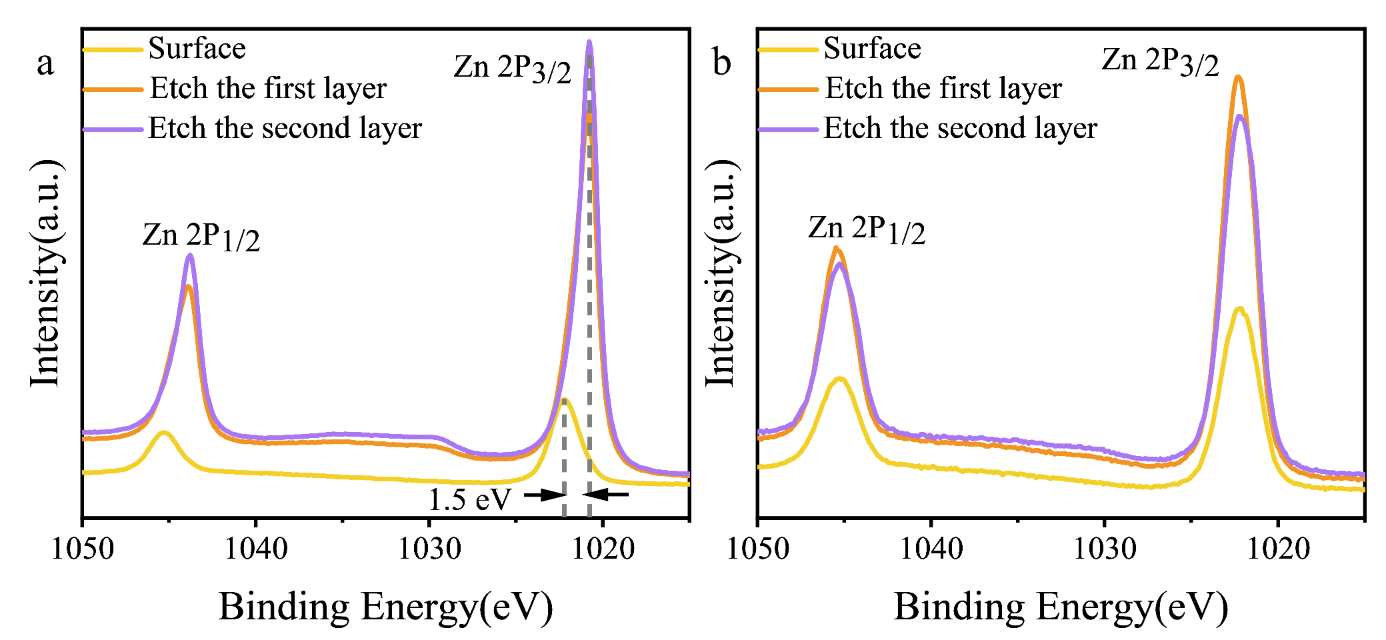


Fig. S16 Depth profiling of the XPS of Zn2p orbitals of Zn electrodes in (a) APM-BE (b) BE


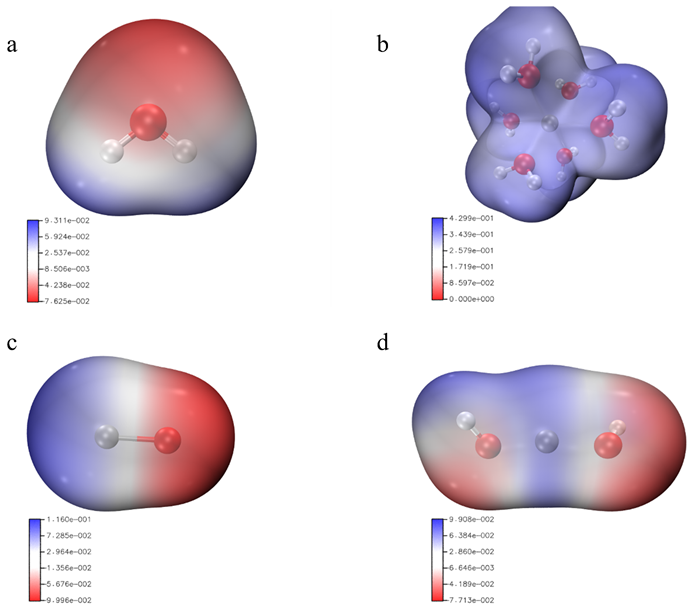


Fig. S17 Electron cloud density diagrams for (a) H_2_O, (b) Zn(H_2_O)_6_^2+^, (c) ZnO and (d)Zn(OH)_2_


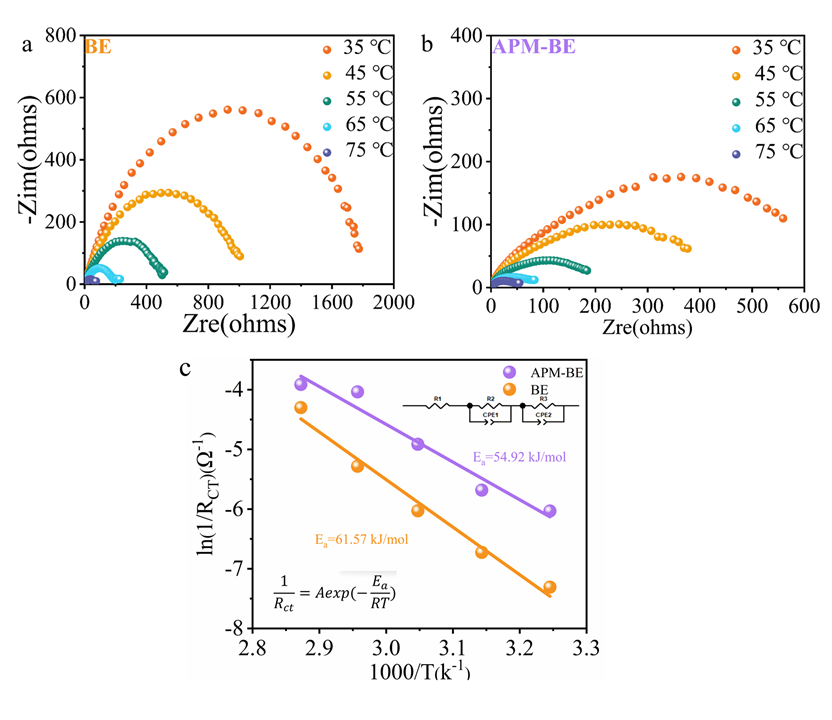


Fig. S18 EIS of Zn‖Zn symmetric cells in (a) APM-BE (b) BE at different temperatures (c) Corresponding Arrhenius curves and comparison of desolvation energies of different electrolyte systems.Arrhenius curves of the Zn electrode for both electrolyte systems, derived from the EIS of the symmetric cell at various temperatures. The EIS data are fitted, and the resulting straight line is derived from Eq. 2


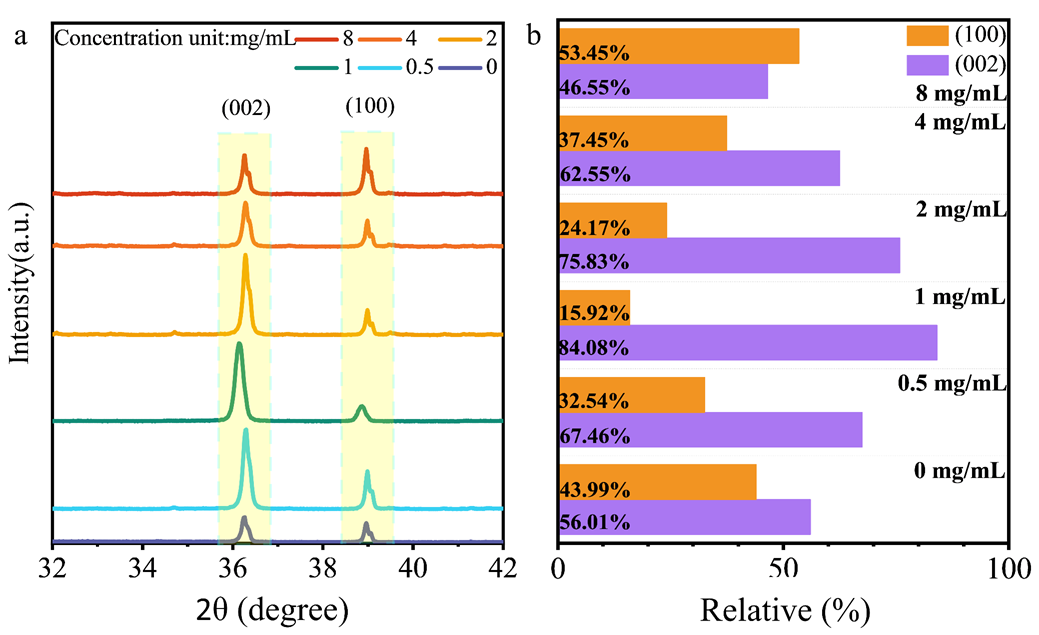


Fig. S19 (a) XRD patterns of Zn electrodes with different electrolytes after 20 cycles at 5 mA cm^-2^-5 mAh cm^-2^. (b) Percentage of (100) and (002) crystalline surfaces of Zn electrodes corresponding to different electrolytes


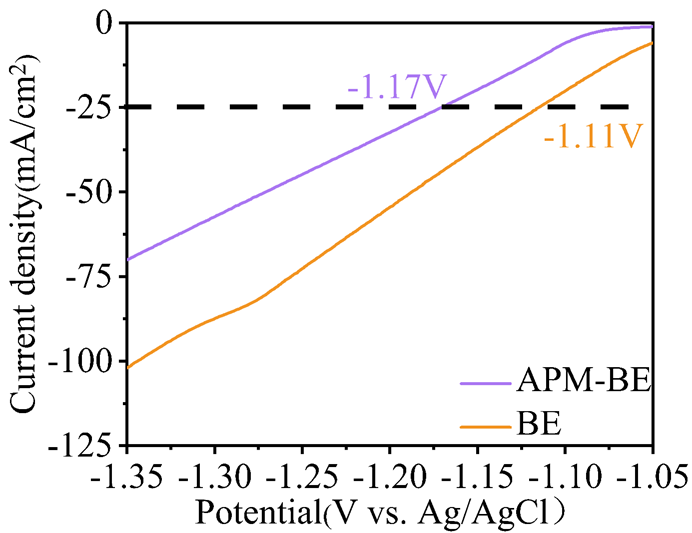


Fig. S20 LSV curves of Zn‖Ti asymmetric -cells with different electrolytes


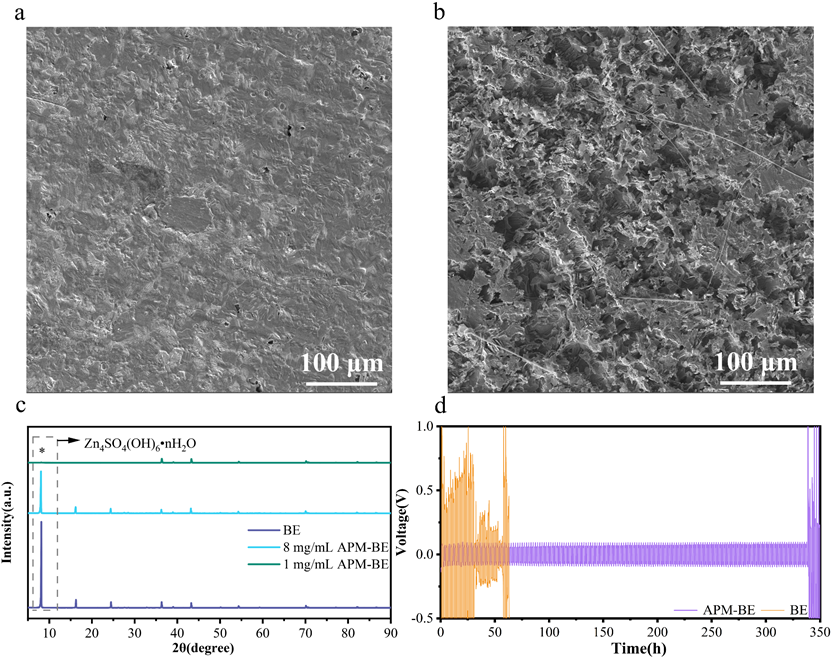


Fig. S21 SEM images of the (a) 1 mg/mL APM-BE (b) BE corresponding to the Zn electrode. (c) XRD patterns of Zn foils immersed in different electrolytes for 15 days.(d) Cycle test using them and with BE assembly as Zn‖Zn symmetric cells

**
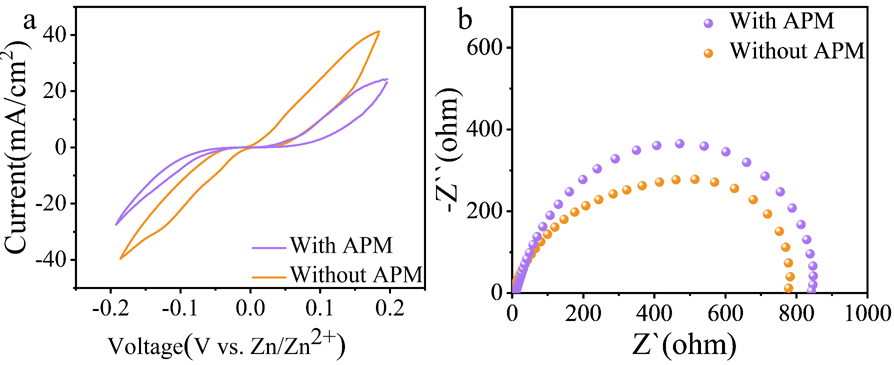
**

Fig. S22 (a) CV and (b) EIS testing of Zn‖Zn symmetric cells with different electrolytes


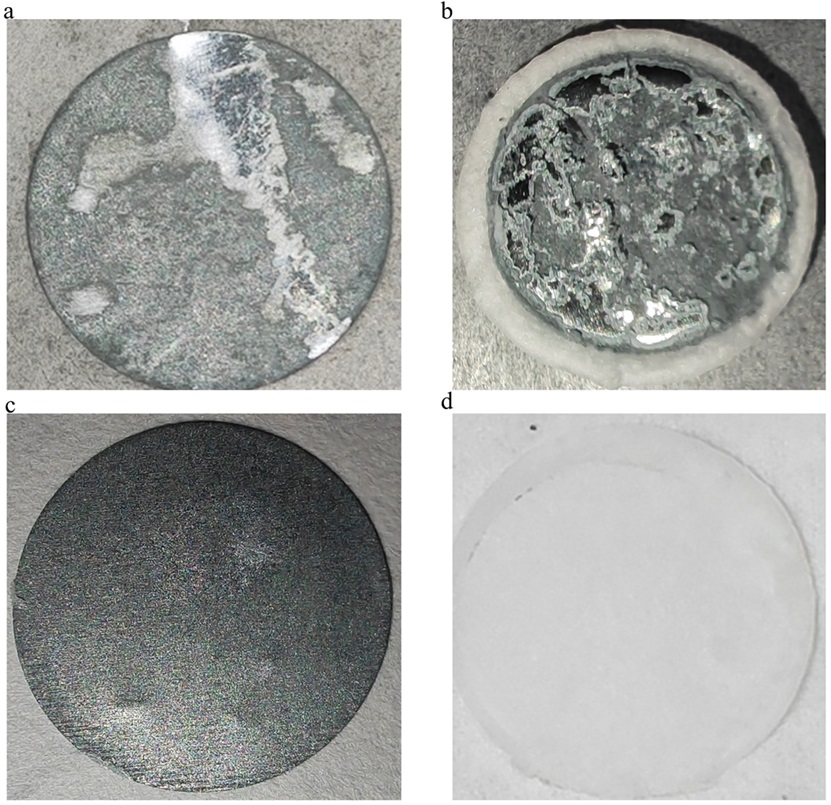


Fig. S23 Digital image of Zn electrode with glass fibre diaphragm using (a-b) BE (c-d) APM-BE after 50 cycles at 5 mA cm^-2^-5 mAh cm^-2^


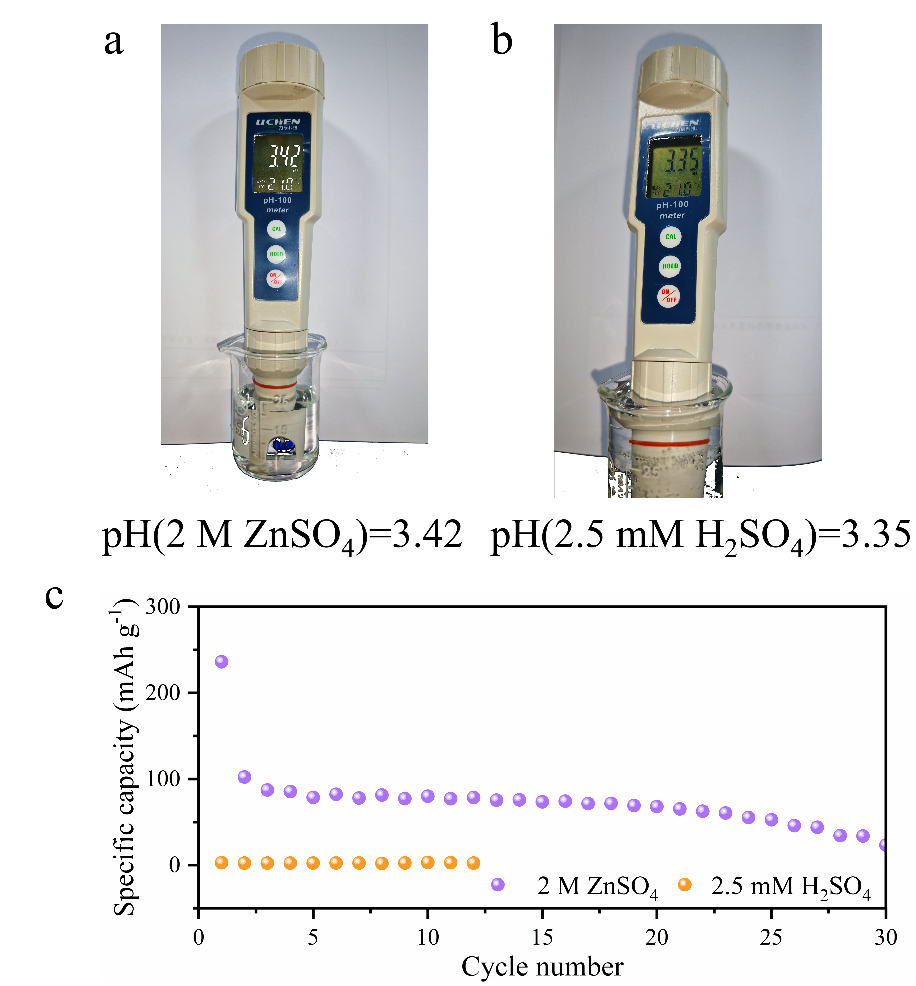


Fig. S24 (a) pH test of 2 M ZnSO_4_ and (b) 2.5 mM H_2_SO_4_. Cycling performance of ZnO||Zn coin cells at 1 A g^-1^ using different electrolytes





Fig. S25 DSC curves of different electrolytes


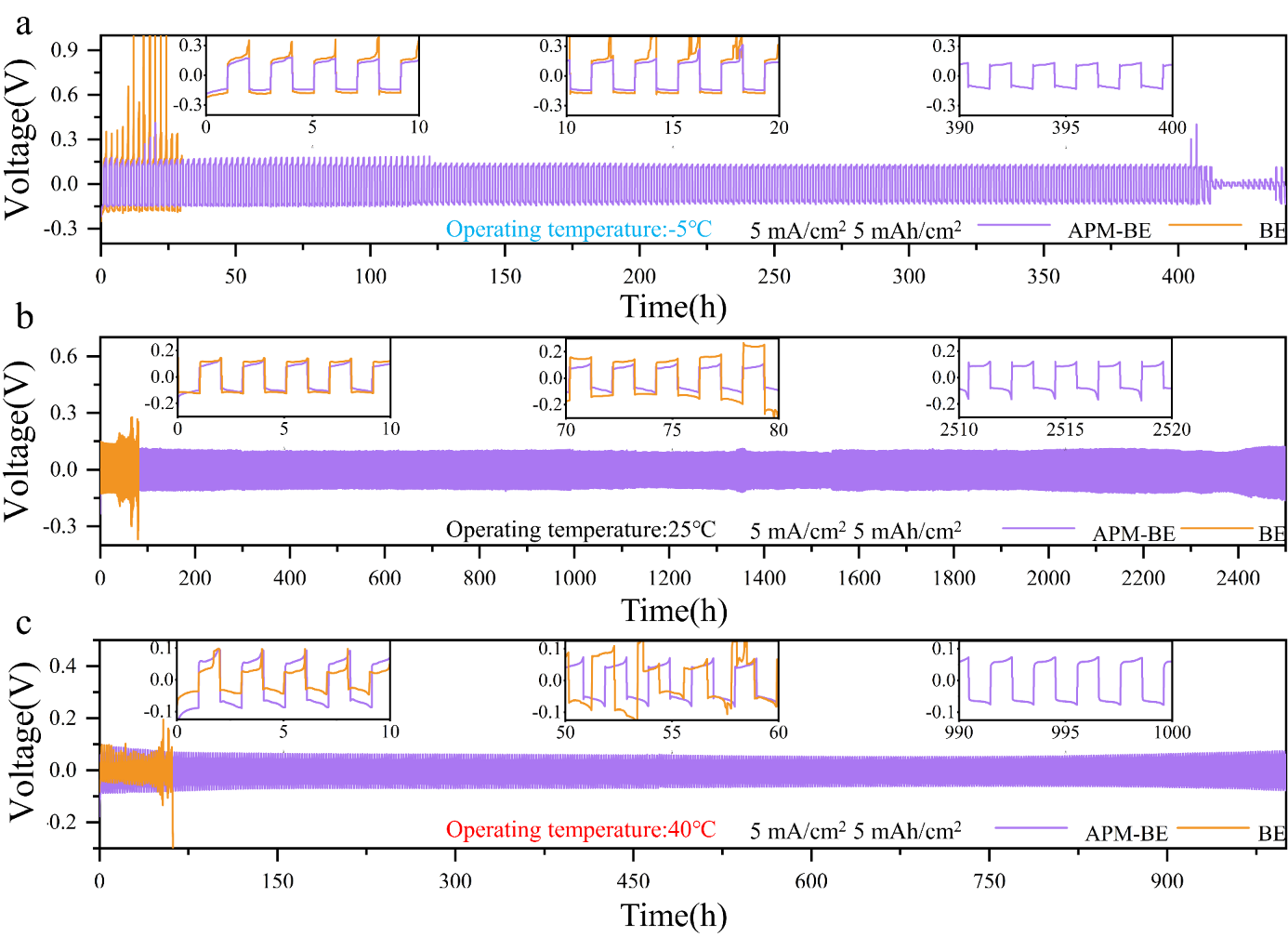


Fig. S26 Cycle test of Zn‖Zn symmetric cells at different test temperatures at 5 mA cm^-2^ 5 mAh cm^-2^


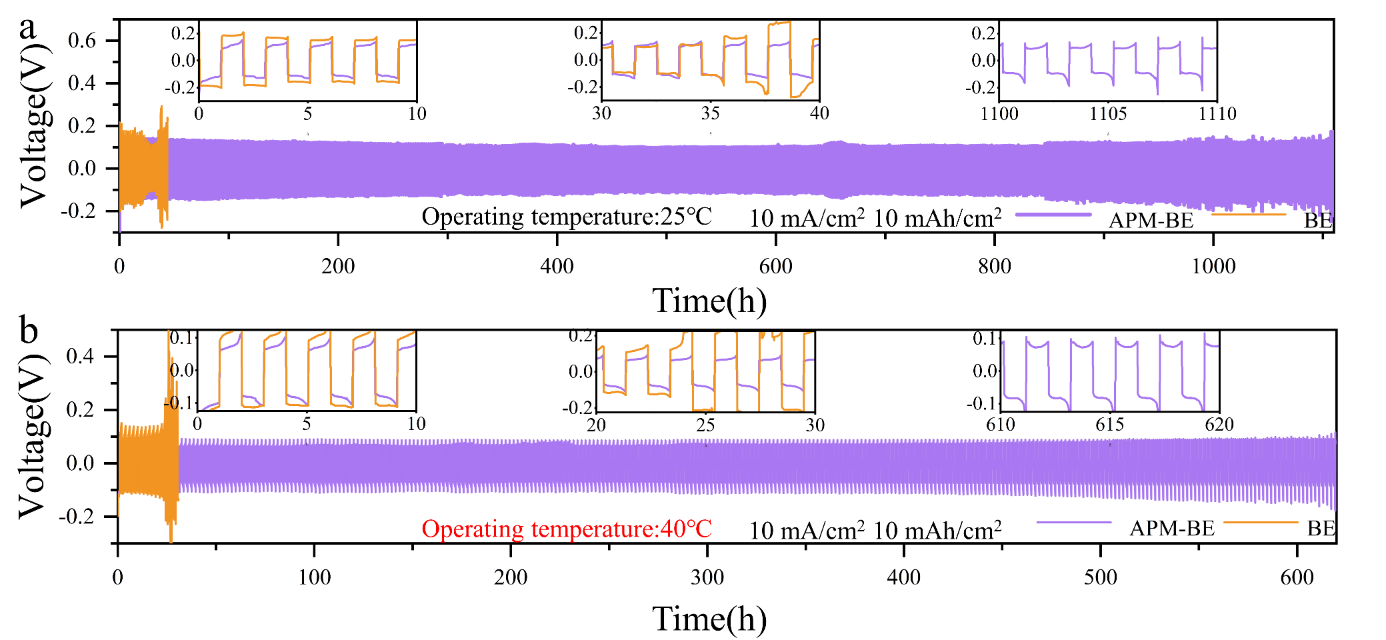


Fig. S27 Cycle test of Zn‖Zn symmetric cells at different test temperatures at 10 mA cm^-2^ 10 mAh cm^-2^


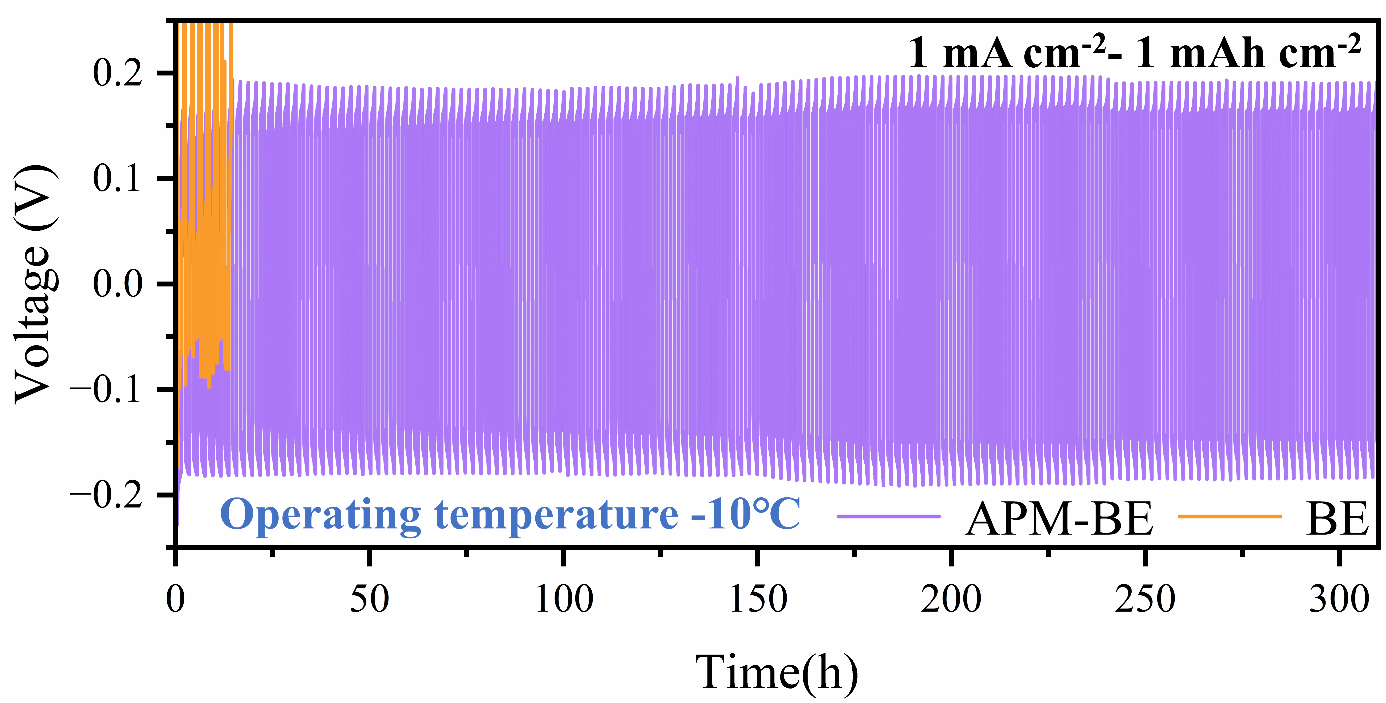


Fig. S28 Cycle test of Zn‖Zn symmetric cells at -10℃ at 1 mA cm^-2^ 1 mAh cm^-2^


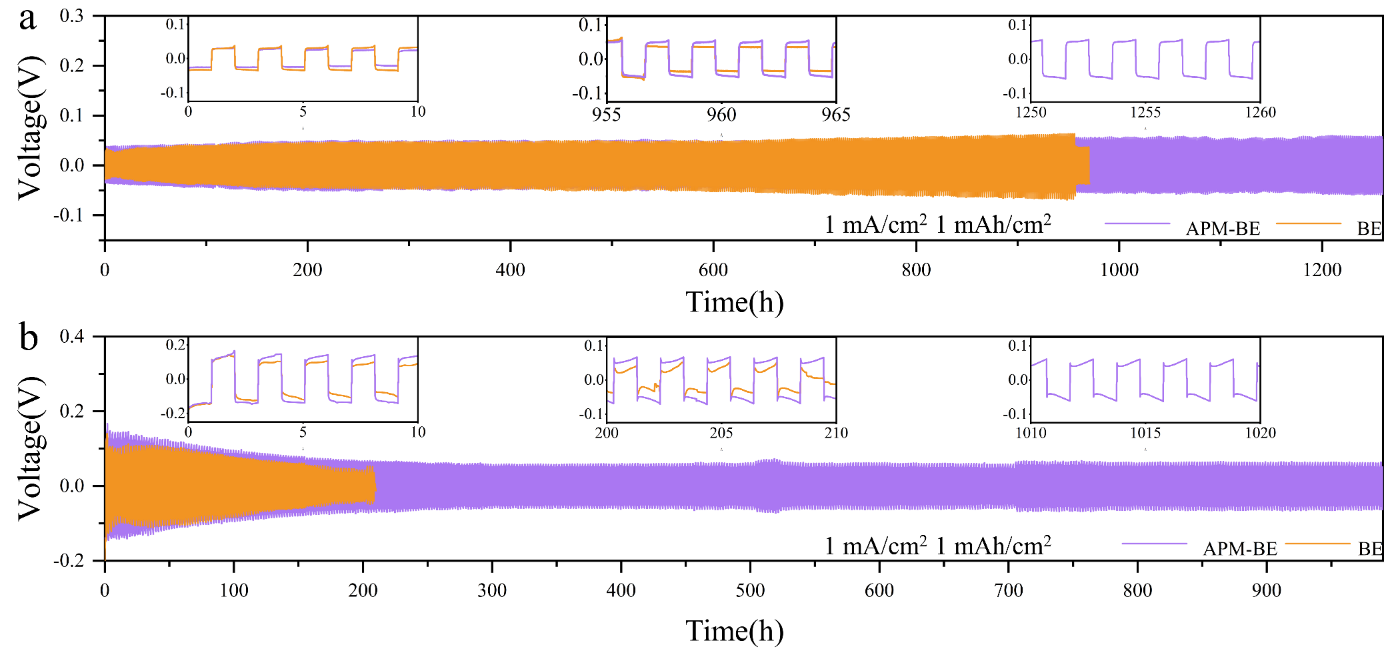


Fig. S29 Cycle test of a Zn‖Zn symmetric cells using (a) ZnBr_2_ and (b) Zn(OTF)_2_ as electrolytes at 1 mA cm^-2^-1 mAh cm^-2^


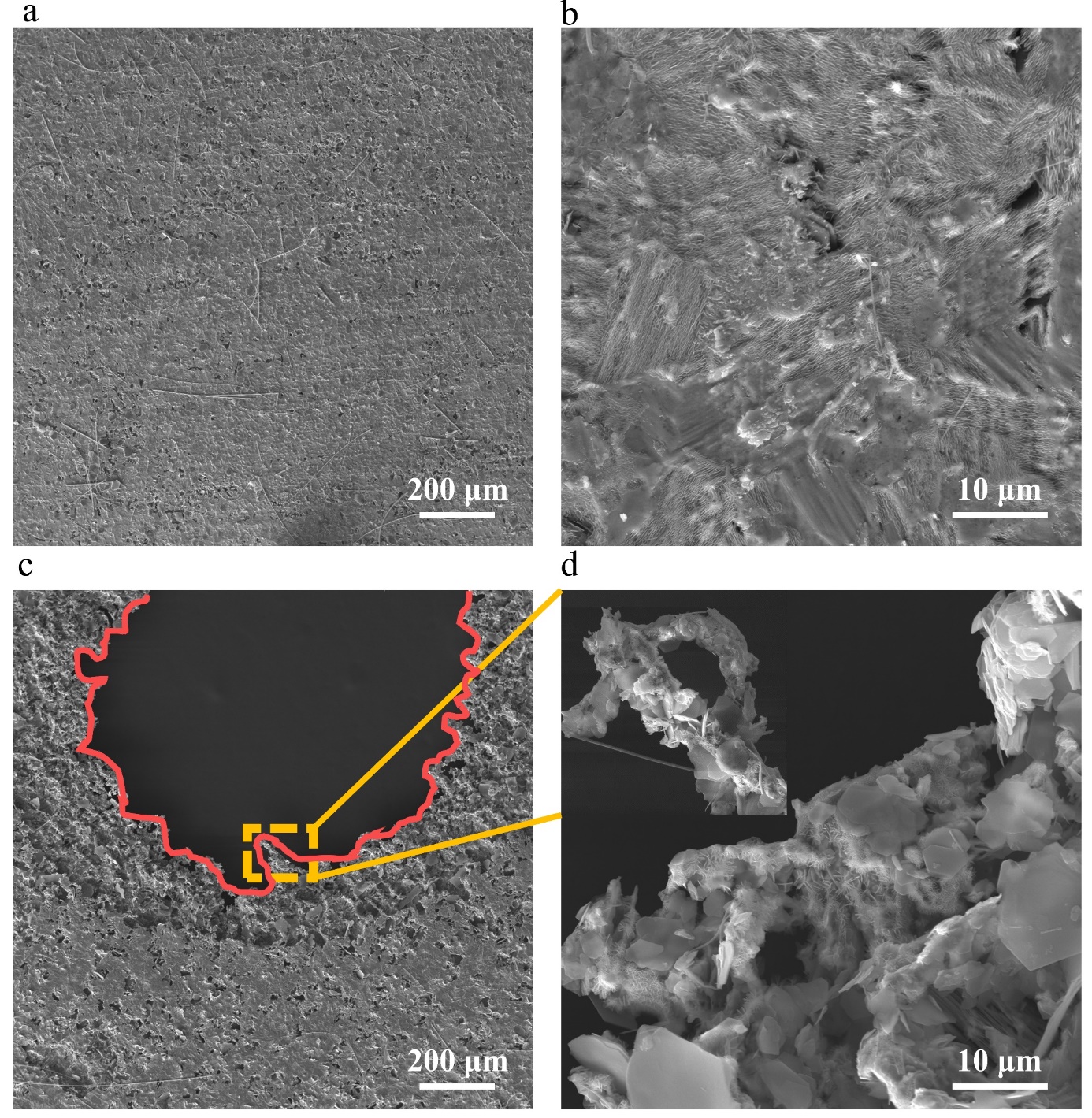


Fig. S30 SEM images of Zn electrodes in a Zn‖Zn symmetric cells with (a-b) APM-BE (c-d) BE after the first cycle at 25 mA cm^-2^-25 mAh cm^-2^


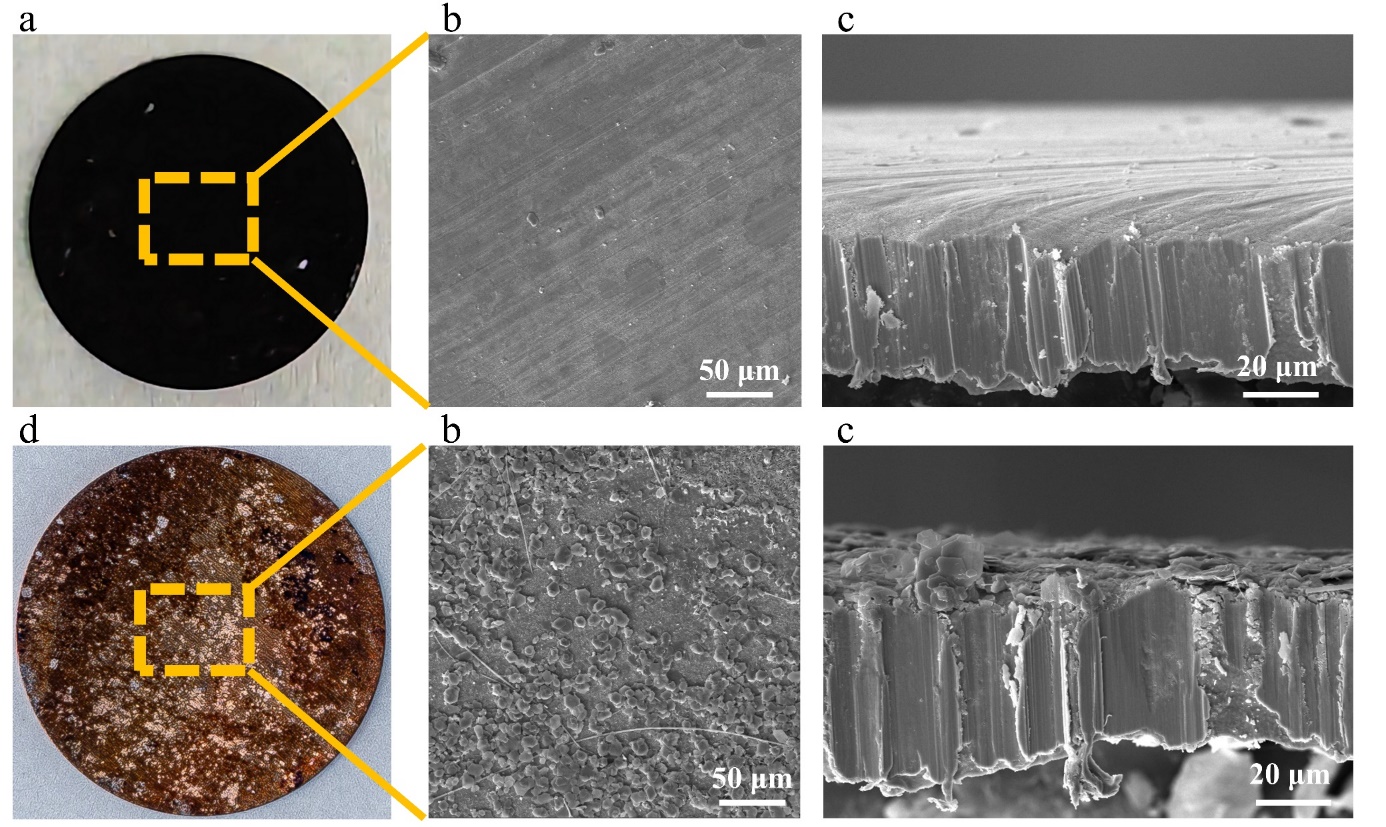


Fig. S31 Digital photographs and SEM images of Cu electrodes in a Zn‖Cu asymmetric -cells using (a-c) APM-BE (d-f) BE after 100 cycles at 1 mA cm^-2^-1 mAh cm^-2^





Fig. S32 Voltage profiles of Zn‖Cu asymmetric cells for the first cycle in different electrolytes


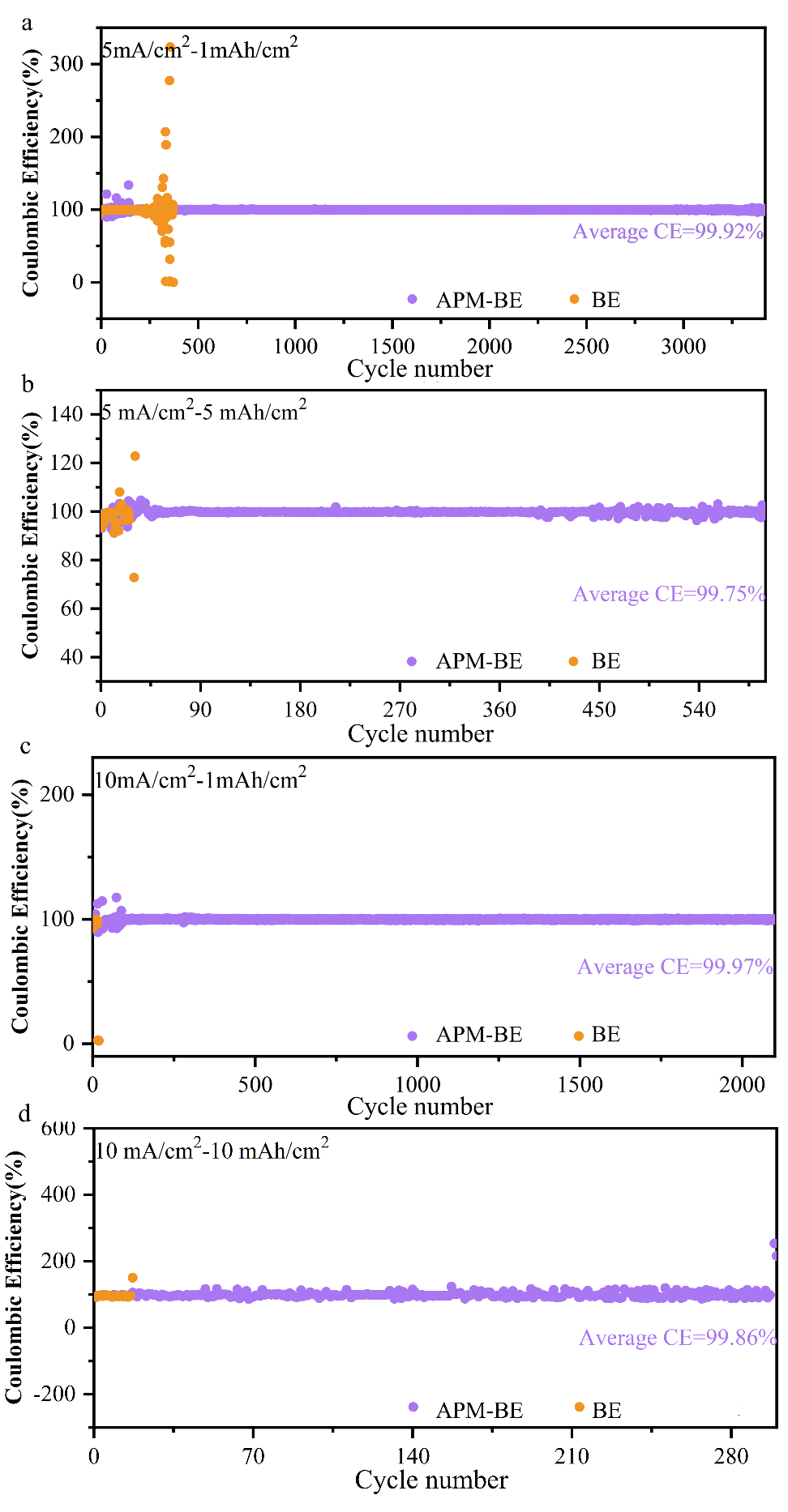


Fig. S33. Coulombic efficiency(CE) test for a Zn‖Cu asymmetric cells at (a) 5 mA cm^-2^-1 mAh cm^-2^, (b) 5 mA cm^-2^-5 mAh cm^-2^, (c) 10 mA cm^-2^-1 mAh cm^-2^, (d) 10 mA cm^-2^-10 mAh cm^-2^


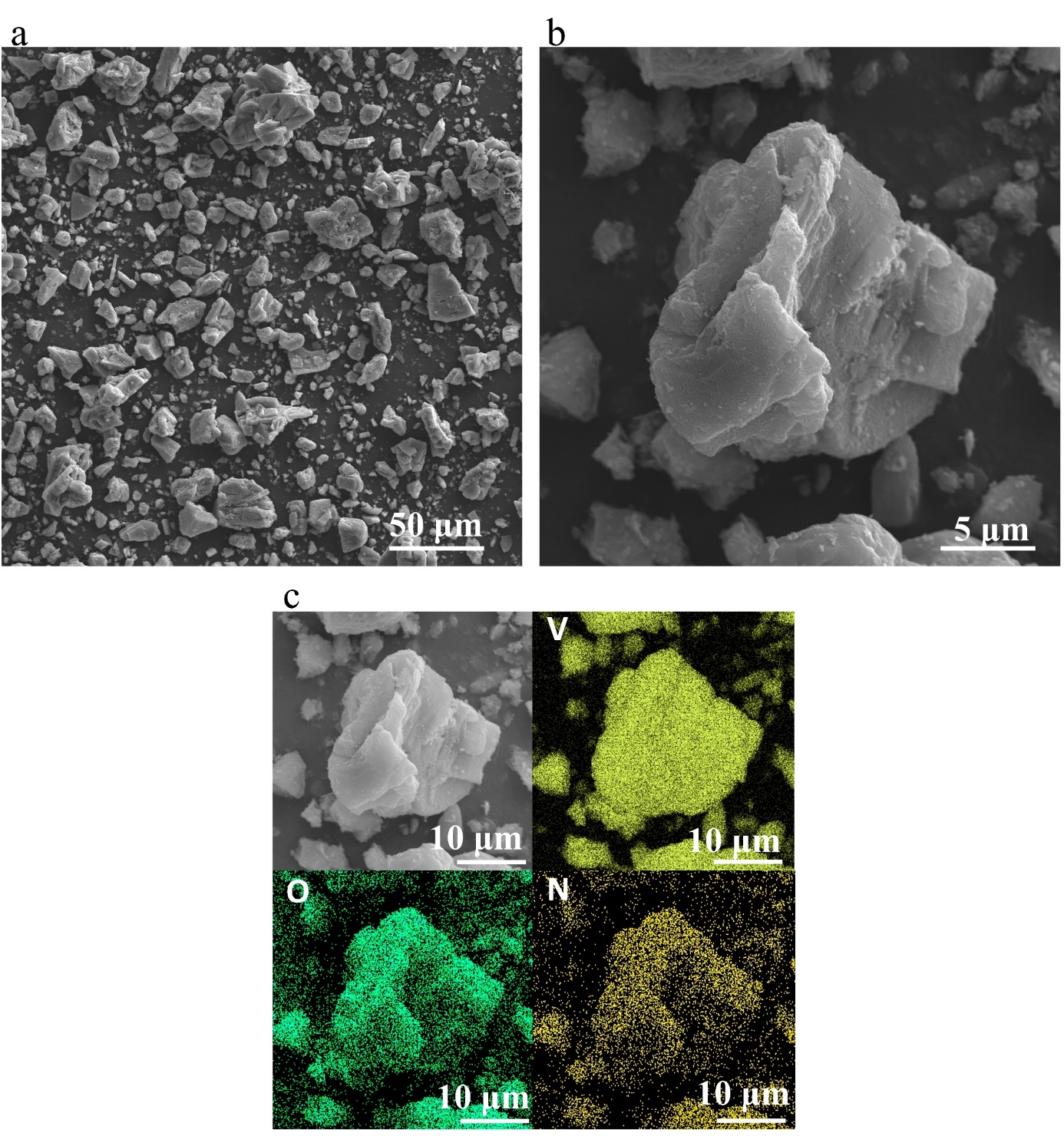


Fig. S34 (a-b) SEM image of NH^4+^-V_2_O_5_ material. (c) EDS image of material


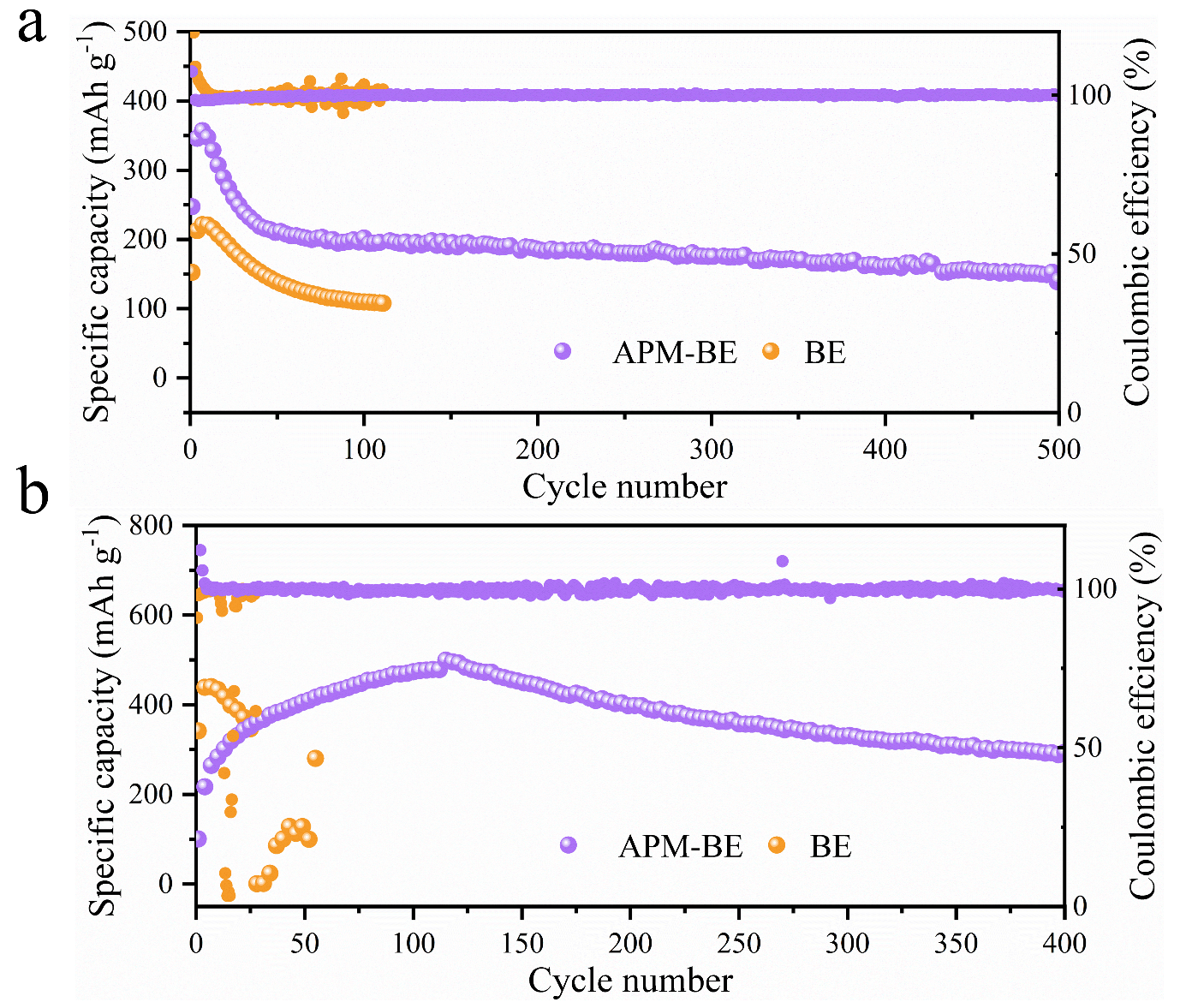


Fig. S35 Full-cell cycling performance at 1 A/g under (a) -5°C and (b) 40°C





Fig. S36 Cycling performance of the full battery with N/P = 1.95 at 1 A/g

|  |
| --- |

|  |
| --- |


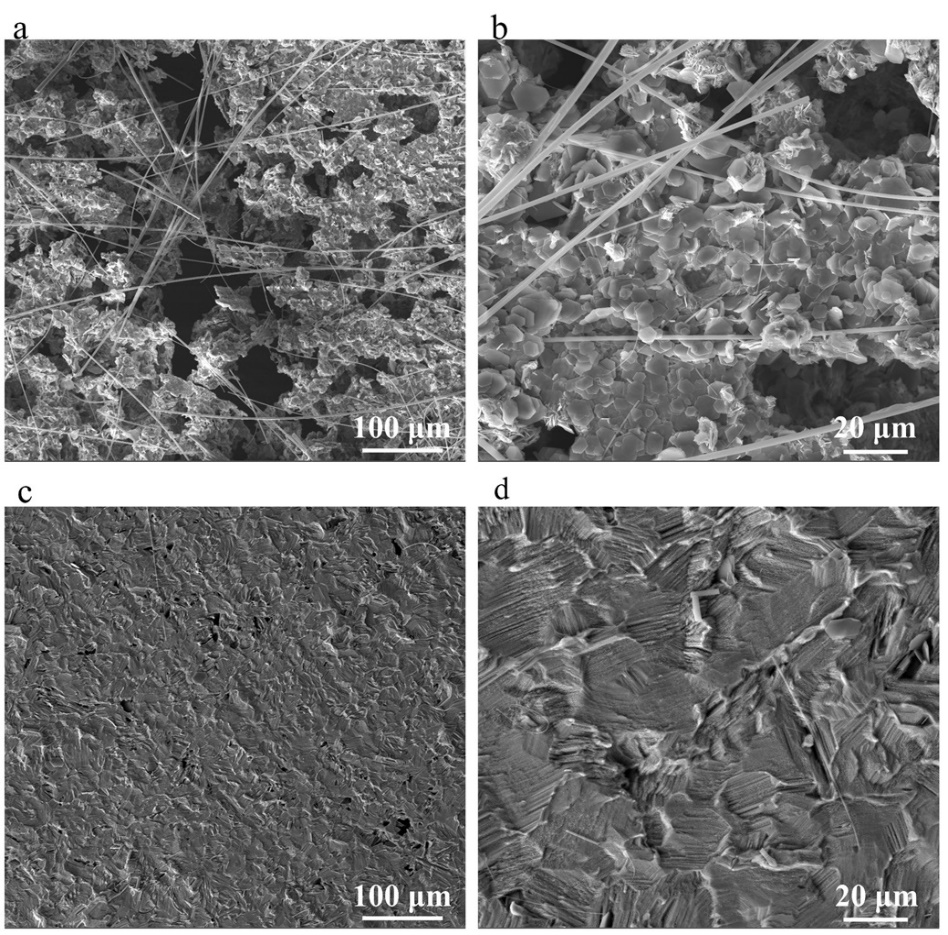


Fig. S37 SEM images of Zn electrodes after full cell cycling to failure using (a-b) BE (c-d) APM-BE at 10 A/g

Table S1. Key results of adsorption energy calculation process.

| Energy/eV | Zn100-H_2_O | Zn100-Apartame-1 | Zn100-Apartame-2 | Zn002-H_2_O | Zn002-Apartame-1 | Zn002-Apartame-2 |
| --- | --- | --- | --- | --- | --- | --- |
| G_1_  (Zn) | -186.357 | -186.357 | -186.357 | -197.186 | -197.186 | -197.186 |
| G_2_  (Isolated molecule) | -14.226 | -243.436 | -243.436 | -14.226 | -243.436 | -243.436 |
| G_3_  (Absorbed Model) | -194.996 | -424.475 | -423.489 | -204.084 | -433.578 | -433.446 |
| ΔE  [G_3_-(G_1_+G_2_)] | 5.587 | 5.318 | 6.304 | 7.328 | 7.044 | 7.176 |

|  |
| --- |
|  |
|  |
|  |

Table S2 Calculated Gibbs free energies and corrections for different molecules

|  | Free Energie (Hartree) | Correction (Hartree) |
| --- | --- | --- |
| Zn(OH)_2_ | -1930.8 | -0.001524 |
| ZnO | -1854.12 | -0.020379 |
| APM | -1030.01 | 0.269643 |
| H_2_O | -76.3232 | 0.003287 |
| APM- H_2_O | -1106.46 | 0.293325 |

**Table S3** Performance comparison of Zn‖Zn symmetric cells with other strategies reported recently

| Stabilization  strategies | Current density/Areal  capacity (mA cm^-2^ /mAh cm^-2^) | Cycling life(h) | Cumulative deposition  capacity (Ah cm^-2^) |
| --- | --- | --- | --- |
| DMAC and TMP additive [S1] | 5/5 | 1600 | 4 |
| 2-Butanone additive [S2] | 5/5 | 600 | 1.5 |
| Imidazolidinyl urea [S3] | 1/0.5 | 1500 | 0.75 |
| Cysteine self-assembled protective layer [S4] | 1/0.5 | 2500 | 1.25 |
| Theophylline additive [S5] | 10/10 | 650 | 3.25 |
| Acesulfame-K additive [S6] | 5/2.5 | 1600 | 4 |
| AMPS additive [S7] | 10/10 | 890 | 4.45 |
| TMP additive [S8] | 5/1 | 530 | 1.325 |
| DASS additive [S9] | 1/1 | 2400 | 1.2 |
| FCNF Diaphragm [S10] | 10/2 | 600 | 3 |
| Ti_3_C_2_T_x_ MXene modified GF septa [S11] | 1/1 | 1180 | 0.59 |
| NTA additive [S12] | 5/0.5 | 2100 | 5.25 |
| CTAB additive [S13] | 2/1 | 2000 | 2 |
| Artificial coating of cyclised polyacrylonitrile (cPAN) [S14] | 0.5/0.5 | 600 | 0.15 |
| Selective etching of reactive (002) zinc surfaces in plain zinc foils [S15] | 20/10 | 400 | 4 |
| Alginic acid (SA) additive [S16] | 0.5/0.5 | 1000 | 0.25 |
| Dextran additive [S17] | 10/10 | 800 | 4 |
| Carbamide additive [S18] | 5/1 | 1100 | 2.75 |
| This work | 1/1 | 10330 | 5.165 |
|  | 5/5 | 2520 | 6.3 |
|  | 10/10 | 1110 | 5.55 |
|  | 25/25 | 160 | 2 |

**Supplementary References**

1. Y. Wang, Z. Wang, W.K. Pang, W. Lie, J.A. Yuwono et al., Solvent control of water O-H bonds for highly reversible zinc ion batteries. Nat. Commun. **14**(1), 2720 (2023). <https://doi.org/10.1038/s41467-023-38384-x>
2. X. Shi, J. Xie, J. Wang, S. Xie, Z. Yang et al., A weakly solvating electrolyte towards practical rechargeable aqueous zinc-ion batteries. Nat. Commun. **15**(1), 302 (2024). <https://doi.org/10.1038/s41467-023-44615-y>
3. X. Wang, Y. Ying, X. Li, S. Chen, G. Gao et al., Preferred planar crystal growth and uniform solid electrolyte interfaces enabled by anion receptors for stable aqueous Zn batteries. Energy Environ. Sci. **16**(10), 4572–4583 (2023). <https://doi.org/10.1039/d3ee01580g>
4. D. Li, Y. Tang, S. Liang, B. Lu, G. Chen et al., Self-assembled multilayers direct a buffer interphase for long-life aqueous zinc-ion batteries. Energy Environ. Sci. **16**(8), 3381–3390 (2023). <https://doi.org/10.1039/d3ee01098h>
5. Z. Cheng, K. Wang, J. Fu, F. Mo, P. Lu et al., Texture exposure of unconventional (101)Zn facet: enabling dendrite-free Zn deposition on metallic zinc anodes. Adv. Energy Mater. **14**(16), 2304003 (2024). <https://doi.org/10.1002/aenm.202304003>
6. P. Li, J. Zhang, Y. Chen, L. Zhang, Z. Zhao et al., Interfacial H_2_O structure matters: realizing stable zinc anodes with trace acesulfame-K in aqueous electrolyte. Adv. Funct. Mater. **34**(27), 2316605 (2024). <https://doi.org/10.1002/adfm.202316605>
7. T. Long, Q.-Y. Zhao, G.-Y. Yin, P.-X. Xie, S. Liu et al., Regulating interfacial ion adsorption for smooth and durable zinc cycling at high area capacity. Adv. Funct. Mater. **34**(24), 2315539 (2024). <https://doi.org/10.1002/adfm.202315539>
8. J. Zhou, F. Wu, Y. Mei, W. Ma, L. Li et al., Highly stable aqueous/organic hybrid zinc-ion batteries based on a synergistic cathode/anode interface engineering. ACS Nano **18**(1), 839–848 (2024). <https://doi.org/10.1021/acsnano.3c09419>
9. J. Cao, Y. Sun, D. Zhang, D. Luo, L. Zhang et al., Interfacial double-coordination effect guiding uniform electrodeposition for reversible zinc metal anode. Adv. Energy Mater. **14**(2), 2302770 (2024). <https://doi.org/10.1002/aenm.202302770>
10. Y. Li, X. Peng, X. Li, H. Duan, S. Xie et al., Functional ultrathin separators proactively stabilizing zinc anodes for zinc-based energy storage. Adv. Mater. **35**(18), 2300019 (2023). <https://doi.org/10.1002/adma.202300019>
11. Y. Su, B. Liu, Q. Zhang, J. Peng, C. Wei et al., Printing-scalable Ti_3_C_2_T*_x_* MXene-decorated Janus separator with expedited Zn^2+^ flux toward stabilized Zn anodes. Adv. Funct. Mater. **32**(32), 2204306 (2022). <https://doi.org/10.1002/adfm.202204306>
12. Z. Liang, C. Li, D. Zuo, L. Zeng, T. Ling et al., Achieving stable Zn metal anode through novel interface design with multifunctional electrolyte additive. Energy Storage Mater. **63**, 102980 (2023). <https://doi.org/10.1016/j.ensm.2023.102980>
13. Z. Liu, R. Wang, Y. Gao, S. Zhang, J. Wan et al., Low-cost multi-function electrolyte additive enabling highly stable interfacial chemical environment for highly reversible aqueous zinc ion batteries. Adv. Funct. Mater. **33**(49), 2308463 (2023). <https://doi.org/10.1002/adfm.202308463>
14. J. Yang, S. Wang, L. Du, S. Bi, J. Zhu et al., Thermal-cyclized polyacrylonitrile artificial protective layers toward stable zinc anodes for aqueous zinc-based batteries. Adv. Funct. Mater. **34**(21), 2314426 (2024). <https://doi.org/10.1002/adfm.202314426>
15. D. Xu, B. Chen, X. Ren, C. Han, Z. Chang et al., Selectively etching-off the highly reactive (002) Zn facet enables highly efficient aqueous zinc-metal batteries. Energy Environ. Sci. **17**(2), 642–654 (2024). <https://doi.org/10.1039/d3ee02522e>
16. H. Dong, X. Hu, R. Liu, M. Ouyang, H. He et al., Bio-inspired polyanionic electrolytes for highly stable zinc-ion batteries. Angew. Chem. Int. Ed. **62**(41), e202311268 (2023). <https://doi.org/10.1002/anie.202311268>
17. J. Li, Z. Guo, J. Wu, Z. Zheng, Z. Yu et al., Dextran: a multifunctional and universal electrolyte additive for aqueous Zn ion batteries. Adv. Energy Mater. **13**(37), 2301743 (2023). <https://doi.org/10.1002/aenm.202301743>
18. Z. Wang, J. Diao, J.N. Burrow, K.K. Reimund, N. Katyal et al., Urea-modified ternary aqueous electrolyte with tuned intermolecular interactions and confined water activity for high-stability and high-voltage zinc-ion batteries. Adv. Funct. Mater. **33**(48), 2304791 (2023). <https://doi.org/10.1002/adfm.202304791>
